# Streptolysin S targets the sodium-bicarbonate cotransporter NBCn1 to induce inflammation and cytotoxicity in human keratinocytes during Group A Streptococcal infection

**DOI:** 10.3389/fcimb.2022.1002230

**Published:** 2022-11-01

**Authors:** Daniel E. Hammers, Deborah L. Donahue, Zachary D. Tucker, Brandon L. Ashfeld, Victoria A. Ploplis, Francis J. Castellino, Shaun W. Lee

**Affiliations:** ^1^ Department of Biological Sciences, University of Notre Dame, Notre Dame, IN, United States; ^2^ Eck Institute for Global Health, University of Notre Dame, Notre Dame, IN, United States; ^3^ Department of Chemistry and Biochemistry, University of Notre Dame, Notre Dame, IN, United States; ^4^ William Myron (W. M.) Keck Center for Transgene Research, University of Notre Dame, Notre Dame, IN, United States

**Keywords:** group A streptococcus, streptolysin S, skin infection, intracellular pH, bacteriocin

## Abstract

Group A *Streptococcus* (GAS, *Streptococcus pyogenes*) is a Gram-positive human pathogen that employs several secreted and surface-bound virulence factors to manipulate its environment, allowing it to cause a variety of disease outcomes. One such virulence factor is Streptolysin S (SLS), a ribosomally-produced peptide toxin that undergoes extensive post-translational modifications. The activity of SLS has been studied for over 100 years owing to its rapid and potent ability to lyse red blood cells, and the toxin has been shown to play a major role in GAS virulence *in vivo*. We have previously demonstrated that SLS induces hemolysis by targeting the chloride-bicarbonate exchanger Band 3 in erythrocytes, indicating that SLS is capable of targeting host proteins to promote cell lysis. However, the possibility that SLS has additional protein targets in other cell types, such as keratinocytes, has not been explored. Here, we use bioinformatics analysis and chemical inhibition studies to demonstrate that SLS targets the electroneutral sodium-bicarbonate cotransporter NBCn1 in keratinocytes during GAS infection. SLS induces NF-κB activation and host cytotoxicity in human keratinocytes, and these processes can be mitigated by treating keratinocytes with the sodium-bicarbonate cotransport inhibitor S0859. Furthermore, treating keratinocytes with SLS disrupts the ability of host cells to regulate their intracellular pH, and this can be monitored in real time using the pH-sensitive dye pHrodo Red AM in live imaging studies. These results demonstrate that SLS is a multifunctional bacterial toxin that GAS uses in numerous context-dependent ways to promote host cell cytotoxicity and increase disease severity. Studies to elucidate additional host targets of SLS have the potential to impact the development of therapeutics for severe GAS infections.

## Introduction

Group A *Streptococcus* (GAS, *Streptococcus pyogenes*) is a Gram-positive, human-associated pathogen that plays an important role in human health. GAS commonly colonizes the skin and mucosal surfaces, where it is usually responsible for mild and often self-limiting infections including pharyngitis, impetigo, and cellulitis (Cunningham, 2000; Carapetis et al., 2005; Henningham et al., 2012; Walker et al., 2014). These mild infections are highly prevalent worldwide, with approximately 600 million annual cases of GAS-mediated pharyngitis alone (Carapetis et al., 2005). In addition to these mild infections, GAS is also able to cause much more severe and life-threatening infections and post-infectious sequelae. For example, inadequate treatment of mild GAS infections can lead to rheumatic heart disease, which is the leading cause of GAS-induced mortality (Carapetis et al., 2005; Zuhlke et al., 2017). GAS is also capable of disseminating beyond the skin and mucosal surfaces, leading to serious and invasive disease manifestations including necrotizing fasciitis and *Streptococcal* toxic shock syndrome (STSS) (Cunningham, 2000; Carapetis et al., 2005; Waddington et al., 2014; Walker et al., 2014; Liu and Lei, 2018). Altogether, the World Health Organization (WHO) has estimated that GAS is responsible for at least 1.78 million new cases of severe GAS disease and 500,000 deaths each year, demonstrating that GAS is a major human pathogen of significant concern (Carapetis et al., 2005).

GAS employs a wide variety of secreted and surface-bound virulence factors to manipulate its environment to cause its assortment of disease outcomes, including the peptide toxin Streptolysin S (SLS) (Cunningham, 2000; Walker et al., 2014; Barnett et al., 2015). SLS is a small (~2.7 kDa), non-immunogenic, post-translationally modified peptide that was first identified as a distinct GAS hemolysin by EW Todd in 1938, setting the stage for the study of hemolysins as major players in GAS pathogenesis (Todd, 1938). It is encoded by the SLS-associated gene (*sag*) operon, which comprises the SagA protoxin, a series of enzymes involved in heterocycle formations as post-translational modifications, and proteins involved in self-immunity and export of the mature peptide to the environment (Datta et al., 2005; Mitchell et al., 2009). The post-translational conversion of cysteine and serine residues to thiazole and oxazole heterocycles allows SLS to be classified as a thiazole-oxazole modified microcin (TOMM), and the *sag* operon responsible for the production of mature SLS is conserved across many bacterial species, with other examples including the Microcin B17 (MccB17) operon in *Escherichia coli* and the Listeriolysin S (LLS) operon in *Listeria monocytogenes* (Cotter et al., 2008; Lee et al., 2008; Molloy et al., 2014). The heterocylization of serine, threonine, and cysteine residues in the TOMM family allows for many of these peptides to interact with DNA, RNA, and proteins in ways that they would otherwise be unable to, when in their unmodified states (Roy et al., 1999). Although residues of SagA necessary for substrate recognition and maturation by SagBCD have been identified (Mitchell et al., 2009), attempts to purify and elucidate the mature structure of the toxin have been unsuccessful, despite nearly a century of research following the initial identification of SLS as a distinct hemolysin.

SLS is an important GAS virulence factor that plays multiple roles in host-pathogen interactions. It is well-known that SLS is capable of lysing erythrocytes, and it is the primary factor responsible for the β-hemolytic phenotype of GAS grown on blood agar (Duncan and Mason, 1976; Betschel et al., 1998; Nizet et al., 2000). Studies to evaluate the mechanism of SLS-dependent hemolysis have shown that it is a colloid-osmotic process that occurs without the degradation of phospholipids (Okazaki, 1971; Duncan and Mason, 1976). Although SLS has traditionally been viewed as a pore-forming toxin that causes host cell lysis through membrane disruption, recent evidence has demonstrated that SLS induces osmotic imbalance and subsequent hemolysis by targeting the anion exchanger Band 3 (encoded by *SLC4A1*) (Higashi et al., 2016). Other TOMMs have been shown to target proteins, such as the related MccB17 of *E. coli* which binds DNA gyrase (Vizan et al., 1991; Heddle et al., 2001). Additional roles of SLS during host infections are also becoming clear, since the toxin is highly upregulated during invasive GAS infections, and it has been shown that GAS lacking the toxin exhibit significant deficiencies in causing skin lesions and disseminating *in vivo* (Datta et al., 2005; Hirose et al., 2019). SLS has also been shown to contribute to the degradation of epithelial cell junctions to promote invasive disease, induce lysis of multiple nonerythrocytic cell types, and have other significant effects in both *in vivo* and *in vitro* infection models (Ginsburg, 1972; Betschel et al., 1998; Miyoshi-akiyama et al., 2005; Sumitomo et al., 2011; Flaherty et al., 2015; Hirose et al., 2019; Lei et al., 2019). These include the ability of SLS to exacerbate the host inflammatory response, thereby disrupting the ability of the host immune system to clear the infection without over-activation that results in damaging hyperinflammation (Flaherty et al., 2015; Castiglia et al., 2016; Flaherty et al., 2018; von Beek et al., 2019). This allows GAS to accelerate disease progression by inducing the release of pro-inflammatory cytokines and subsequent programmed cell death during infection. Overall, GAS appears to utilize SLS to target specific host factors, causing tissue damage and increased disease severity in multiple ways.

Although Band 3 has been identified as an erythrocyte target for SLS, the possibility that SLS may have additional protein targets in other cell types has not been explored. The idea that bacterial virulence factors could have multiple context-dependent targets is not new; for example, *Staphylococcus aureus* produces alpha toxin, which is able to target a variety of cell types, and can have different effects within the same cell type depending on the concentration of the toxin (Galdiero and Gouaux, 2004; Wilke and Wardenburg, 2010; Powers et al., 2012; Berube and Bubeck Wardenburg, 2013). One such cell type that is particularly relevant for GAS pathogenesis is keratinocytes, which are typically the first cell type encountered by GAS during skin-associated infections such as necrotizing fasciitis. SLS induces a series of signaling responses in human keratinocytes, including the downregulation of pro-survival Akt signaling and the upregulation of p38 MAPK and NF-κB signaling, driving the production of pro-inflammatory mediators and causing programmed cell death (Flaherty et al., 2015; Flaherty et al., 2018). Keratinocytes do not express Band 3, suggesting that SLS may target other proteins in these cells to cause these effects. Here, we use bioinformatic analysis and chemical inhibition studies to demonstrate that SLS targets the electroneutral sodium-bicarbonate cotransporter NBCn1 (encoded by *SLC4A7*) in keratinocytes during GAS infection, disrupting pH homeostasis and causing an inflammatory response resulting in host cell death. These results further support the idea that SLS is a multifunctional bacterial toxin that GAS uses in numerous context-dependent ways to promote host cell cytotoxicity and increase disease severity.

## Materials and methods

### Bacterial cultures and strains

Group A *Streptococcus* (M1T1 strain 5448) was utilized for these experiments. Strains included a wild-type M1T1 5448 strain (referred to here as M1 5448), the isogenic mutant *sagAΔcat* (a gift from V. Nizet, referred to here as the *ΔsagA* strain), and a complemented *sagAΔcat* + *sagA* strain (referred to here as the *ΔsagA* + *sagA* strain). The wild-type and *ΔsagA* strains have been characterized elsewhere (Kansal et al., 2000; Datta et al., 2005). The *ΔsagA* + *sagA* strain was generated in our laboratory, and has also been previously described (Flaherty et al., 2015; Higashi et al., 2016). All GAS strains were grown on Todd-Hewitt (TH, Neogen) media or Group A *Streptococcus*-selective media with 5% sheep blood (BD), and liquid cultures were grown for 16-20 hours at 37° C in TH broth prior to infection experiments. Cultures of the *ΔsagA* + *sagA* strain were carried out along with 5 µg/mL erythromycin for selection.

### Keratinocyte cell culture

The immortalized human keratinocyte cell line HaCaT (a gift from V. Nizet) was used for these studies, and has been described elsewhere (Boukamp et al., 1988). Cells were maintained in Dulbecco’s Modified Eagle Medium (DMEM, Gibco) supplemented with 10% heat-inactivated and fetal bovine serum (FBS, Biowest). Prior to supplementation, the FBS was filter-sterilized (0.22 µm Steriflip filters, Millipore). Cells were grown at 37° C with 5% CO_2_.

### Keratinocyte infections

HaCaT cells were seeded onto either sterilized glass coverslips in 6-well tissue culture-treated plates (Corning) for IF microscopy experiments, 35 mm glass bottom imaging dishes (MatTek) for live imaging experiments, or 24-well tissue culture-treated plates (Eppendorf) for cytotoxicity assays and other keratinocyte treatments. Cells were grown to 80-90% confluency, and were washed with sterile 1x PBS (Gibco) prior to the addition of fresh DMEM + 10% FBS. For infections that were performed with chemical inhibitors, this fresh media contained either 100 µM of the compound, or DMSO as a vehicle control. The final DMSO concentration in all wells was 0.8%. Cells were pre-treated for 1 hour at 37° C with 5% CO_2_. Meanwhile, overnight GAS cultures were centrifuged and re-suspended in fresh TH, and their optical densities were normalized. Following the pretreatment step, HaCaT cells were infected at a multiplicity of infection (MOI) of 10 for cytotoxicity and IF microscopy experiments, and MOI 0.01 for live imaging experiments. Sterile TH was used as an uninfected control. Infections were carried out at 37° C with 5% CO_2_ for the specified times. A summary of the general infection protocol is shown in **Figure S1**.

### Ethidium homodimer cytotoxicity assay

HaCaT cells were infected as described above. Following the infection, supernatants were collected to ensure that any dead, non-adherent cells would be included in the cytotoxicity measurements. Cells remaining on the plate were washed twice with sterile 1x PBS, and washes were pooled with the supernatants. Pooled supernatants and washes were centrifuged at 9,300 x g for 10 minutes to pellet cell debris, and the supernatant was aspirated. This pellet was re-suspended in 4 µM ethidium homodimer 1 (Molecular Probes) in PBS, and was redistributed to a 24-well plate. Cells remaining on the original plate were incubated with the same concentration of ethidium homodimer 1. Both plates were covered and incubated at room temperature with gentle agitation for 30 minutes, and fluorescence was measured on a Synergy H1 Microplate reader set to 528 nm excitation and 617 nm emission, with a cutoff value of 590 nm. To normalize the fluorescence reading to the total number of cells per well, cells were covered again and incubated with 0.1% (wt/vol) Saponin (Sigma) for 20 minutes with gentle agitation, followed by another plate reader measurement using the same settings. The percent membrane permeabilization and corresponding cytotoxicity were calculated using the combined post-infection readings divided by the combined post-Saponin readings for each well.

### SLS preparations

SLS preparations were generated as previously described (Higashi et al., 2016). Briefly, preparations were generated by transferring overnight GAS cultures on TH plates to TH broth, and optical densities were normalized. 5 x 10^7^ CFU/mL of bacteria were mixed in PBS on ice at a final concentration of 1:40, and were incubated for 30 minutes. All subsequent steps were carried out on ice or at 4° C. Following incubation, bacterial suspensions in PBS were centrifuged at 2000 x g for 10 minutes, and the supernatants were filter sterilized using 0.22 µm Steriflip filters (Millipore). Supernatants were concentrated tenfold by centrifugation using a filtration unit with a 3K MWCO (Pall), and were either used immediately for hemolysis assays or stored at -20°C until use.

### Hemolysis assay

Hemolysis assays were performed as previously described (Higashi et al., 2016). Defibrinated whole sheep blood (Lampire) was washed with cold PBS, and was diluted 1:200 in cold PBS. Blood was treated with either DMSO as a vehicle control (final concentration 0.4%) or 50 µM S0859, prior to treatment with SLS preparations at a 1:10 ratio of SLS preparations, PBS, or 10% Triton X-100 (Sigma) in PBS to blood. These mixtures were incubated at 37°C for 1 hour, and were centrifuged at 0.4 x g at 4°C for 10 minutes to pellet intact erythrocytes. Supernatants containing hemoglobin that was released by hemolysis were subjected to absorbance measurements (450 nm), and hemolysis values were calculated by normalizing to the 10% Triton and PBS conditions.

### Bioinformatics and protein expression

A protein BLAST (NCBI) (Altschul et al., 1997) was used to identify proteins with significant homology to a synthetic peptide that corresponded to a putative binding domain for SLS (query sequence: PWRMHIFTIIQVACLVLLWVVRSIK) (Higashi et al., 2016). This sequence was derived from the C-terminal domain of Band 3 from *Ovis aries* (NCBI Reference Sequence XP_004013030.2), which was chosen because these Band 3 – SLS studies were performed with sheep blood (Higashi et al., 2016). The search was limited to human proteins (taxid: 9606). Human NBCn1 (solute carrier family 4 sodium bicarbonate cotransporter member 7, GenBank ACH61959.1) was identified as having high similarity to the query, and was aligned with full-length *Ovis aries* Band 3 using Clustal Omega (Sievers et al., 2011). The Human Protein Atlas (proteinatlas.org) was used to assess expression profiles of selected proteins (Uhlén et al., 2015).

### Immunofluorescence microscopy

HaCaT cells were seeded onto glass coverslips, treated with inhibitors, and infected with GAS as described above. After the infection, cells were washed with cold PBS and fixed in 4% (wt/vol) paraformaldehyde in PBS for 1 hour. Cells were washed with cold PBS and incubated with a blocking buffer containing 1% (wt/vol) normal goat serum (Invitrogen), 2% (vol/vol) Triton X-100 (Dow/Sigma Aldrich), and 0.5% (vol/vol) Tween 20 (Sigma) in PBS for 2 hours at room temperature. Cells were washed with 1x PBS for 20 minutes and incubated with 1:50 of primary antibody in blocking buffer at 4° C overnight. Coverslips were washed in PBS for 90 minutes, and were incubated with 1:200 secondary antibody in blocking buffer for 2 hours at room temperature. Cells were washed with PBS for 1 hour, and were incubated with 1:500 DAPI nuclear stain and 1:500 rhodamine-phalloidin actin stain in blocking buffer for 30 minutes at room temperature. Coverslips were washed in PBS for 45 minutes, and were mounted to glass slides using Fluoromount-G (Southern Biotech). Once set, slides were imaged using an inverted Nikon Eclipse Ti-E microscope with an iXon Ultra 897 electron multiplying charge-coupled device (Andor) or a Neo sCMOS (Andor) using the 60x objective with oil immersion. Images were analyzed and reconstructed using FIJI/ImageJ (NIH).

### Intracellular pH monitoring with pHrodo

Keratinocytes were loaded with pHrodo Red AM (Molecular Probes) according to the manufacturer’s instructions. Briefly, pHrodo Red AM was diluted 10x in PowerLoad concentrate, and this mixture was diluted 100x in Live Cell Imaging Solution (LCIS, Invitrogen). Meanwhile, HaCaT cells were washed 1x with sterile LCIS, and were incubated with the diluted pHrodo dye for 30 minutes while covered at 37°C with 5% CO_2_. Excess dye was aspirated, and cells were washed 2x with sterile LCIS. For real-time live imaging experiments, cells were incubated with pre-warmed DMEM + 10% FBS and infected with GAS at an MOI of 0.01. This lower MOI was used to ensure that cells would not be overwhelmed with bacteria over the time course. Infections were imaged on the Nikon Eclipse Ti-E microscope described above, fitted with an environmental chamber set to maintain 37°C and 5% CO_2_. Images were taken in the DIC and pHrodo (excitation 555 nm, emission 580 nm, exposure 10 ms) every 10 minutes for 8 hours. Images were reconstructed into time-lapse videos using ImageJ. Experiments were performed at least in triplicate for each condition to confirm individual phenotypes, and representative videos are shown.

### Necrotizing skin infection of humanized mice with S0859 treatment

For all experiments, human plasminogen-expressing C57BL/6 [hPg (Tg)] (Sun et al., 2004) mice at 6-10 weeks of age were injected subcutaneously in the flank with 100 µL of GAS (10^7^ CFU) with specified concentrations of S0859 or 0.7% saline as a vehicle control. Experiments were performed in a non-blind setting, with no exclusion of animals. Animals for each experimental group were selected randomly. At the end of each experiment, wound measurements and images were taken, and mice were sacrificed under rodent cocktail anesthesia (9 parts ketamine (100 mg/mL) + 9 parts xylazine (20 mg/mL) + 3 parts acepromazine (10 mg/mL) + 79 parts saline), (body weight x 10) - 50 μL = μL/mouse). Wounds were excised and prepared for determination of bacterial colony-forming units as previously described (Datta et al., 2005). Animal protocols were approved by the Institutional Animal Care and Use Committee at the University of Notre Dame.

Two experimental approaches were used: (1) a single dose of treatment (100 µM S0859 or vehicle) administered at the time of bacterial inoculation. Here, 4 mice per group (wild-type + vehicle, wild-type + 100 µM S0859, ΔsagA + vehicle, ΔsagA + sagA + vehicle) were injected in a single experiment. Mice were monitored and wounds were measured every 24 hours for 72 hours. (2) a dose of treatment (200 µM S0859 or vehicle) administered at the time of bacterial inoculation, followed by additional doses at 4 and 8 hours post-infection. Here, the data from two separate experiments were pooled, using only wild-type + vehicle (total n = 7) and wild-type + 200 µM S0859 (total n = 9) as groups. Wound measurements and images were taken after 24 hours, and mice were sacrificed and prepared as described above.

### Inhibitor compounds, antibodies, and stains

Inhibitor compounds were dissolved in DMSO and used at the indicated concentrations. The N-cyanosulfonamide S0859 (Cayman) and stilbenedisulfonates 4,4’-diisothiocyanatostilbene-2,2’-disulfonate (DIDS, Sigma), 4,4’-diaminostilbene-2,2’-disulfonate (DADS, Sigma), and 4-acetamido-4′-isothiocyanato-stilbene-2,2′-disulfonate (SITS, Sigma) were commercially available, while the DIDS analog 4,4’-ocatanamidostilbene-2,2’-disulfonate (OADS) was synthesized by the Ashfeld group as previously described (Howery et al., 2012). Antibodies to NF-κB (Rb α NF-κB p65, Cell Signaling Technology D14E12, RRID:AB_10859369), rabbit IgG (Gt α Rb IgG AlexaFluor 488, Invitrogen, RRID:AB_143165), and SLC4A7 (Rb α SLC4A7, Fisher PA557433, RRID:AB_2647530) were used at the indicated concentrations. The nuclear stain 4′,6-diamidino-2-phenylindole (DAPI, Cell Signaling Technology) and actin stain rhodamine-phalloidin (Thermo-Fisher) were used at the indicated concentrations.

### Statistical analysis

All experiments were performed at least in triplicate. Statistical analysis was performed using GraphPad Prism 9.0. The similarity of standard deviations between conditions within each experiment and the lack of outlier values led us to assume normal distributions for all data sets. Significance was determined using ANOVA for all experiments in which >2 means were being compared, with *post hoc* Tukey’s HSD tests as necessary (α = 0.05). Significance was reported as follows for all data sets: *: 0.01 < *P* < 0.05, **: 0.001 < *P* < 0.01, ***: 0.0001 < *P* < 0.001, ****: *P* < 0.0001, n.s.: not significant.

## Results

### Treatment with the stilbenedisulfonate DIDS inhibits SLS-dependent cytotoxicity during GAS infection

**Figure 1:**
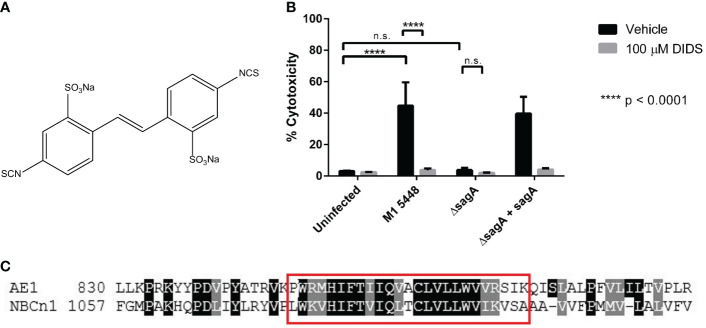
SLS-mediated keratinocyte cytotoxicity involves proteins that are associated with ion transport. **(A)** Chemical structure of the stilbene disulfonate DIDS. **(B)** Keratinocytes were pretreated for 1 hour with 100 µM DIDS, and then were infected with GAS at MOI 10 for 6 hours. Percent cytotoxicity was determined post-infection using an ethidium homodimer assay. DMSO was used as a vehicle control for all infections. Data are presented as mean ± sd, with n = 3 for each condition. **(C)** Band 3 and NBCn1 have a similar region near their C-termini. A protein BLAST was used to identify proteins with similar sequences to the synthetic peptide used by Higashi et al. to inhibit SLS-dependent hemolysis (Higashi et al., 2016). NBCn1 was found to exhibit a high degree of similarity in the region spanned by this peptide. A selection of the alignment between Band 3 (AE1) from *Ovis aries* (NCBI Reference Sequence XP_004013030.3) and human NBCn1 (GenBank ACH61959.1) is shown. The red box denotes the AE1 sequence used to generate the synthetic peptide used as a query. **** = P < 0.0001, n.s. = not significant.

Previous studies have demonstrated that Band 3-mediated hemolysis induced by SLS can be mitigated by chemically treating erythrocytes with 4,4’-diisothiocyanatostilbene-2,2’-disulfonate (DIDS) prior to the application of bacterial supernatants (Higashi et al., 2016). DIDS is a known Band 3 inhibitor (Okubo et al., 1994; Matulef et al., 2008; Arakawa et al., 2015), and this demonstrated that SLS is capable of targeting host proteins to induce cytolysis. The chemical structure of DIDS is shown in **Figure 1A**. Although keratinocytes do not express Band 3, we hypothesized that treating keratinocytes with DIDS during a GAS infection would similarly inhibit SLS-dependent cytotoxicity. Therefore, we performed keratinocyte infections following pre-treatment of HaCaT human keratinocytes with 100 µM DIDS, using DMSO as a vehicle control (**Figure 1**). Following the infection, percent membrane permeabilization corresponding to percent cytotoxicity was assessed using the membrane-impermeable, DNA-intercalating fluorescent dye ethidium homodimer. Keratinocytes were infected for 6 hours at an MOI of 10 based on previous results for optimizing SLS expression in our *in vitro* infection model (Flaherty et al., 2015).

When HaCaT cells were treated with DMSO as a vehicle control, infection with SLS-producing strains of M1T1 5448 GAS (M1 5448, *ΔsagA* + *sagA*) resulted in nearly 50 percent cytotoxicity, which was significantly greater than the cytotoxicity induced by the uninfected control or cells infected with the SLS-deficient *ΔsagA* strain (**Figure 1B**). These data were consistent with previous studies of GAS infections in the HaCaT cell line (Flaherty et al., 2015). Treatment with 100 µM DIDS did not significantly impact cell viability in the uninfected or *ΔsagA* conditions, but the cytotoxicity observed in the SLS-producing conditions was reduced to levels comparable to that of the uninfected control. These results demonstrated that the stilbenedisulfonate DIDS inhibits keratinocyte cytotoxicity in an SLS-dependent manner during GAS infection. In addition, other related stilbene derivatives also inhibited SLS-dependent cytotoxicity in keratinocytes during GAS infection (**Figure S2**). Treatment of keratinocytes with 100 µM each of OADS (4,4’-ocatanamidostilbene-2,2’-disulfonate, **Figure S2A**) and SITS (4-acetamido-4′-isothiocyanato-stilbene-2,2′-disulfonate, **Figure S2B**), but not DADS (4,4’-diaminostilbene-2,2’-disulfonate, **Figure S2C**), inhibited SLS-dependent cytotoxicity in a manner similar to DIDS. DIDS contains two isothiocyanate groups, which are replaced with amines in DADS and alkyl chains connected to the stilbene core through amide linkages in OADS. SITS retains one isothiocyanate from DIDS, while the other is replaced by an acetyl group. This suggested that the isothiocyanate groups of these compounds played a significant role in the inhibition of cytotoxicity, which could be explained by the reported ability of these groups to react with lysine side chains in Band 3 (Okubo et al., 1994).

### NBCn1 displays homology to Band 3 in a putative SLS-binding region

While the inhibition of SLS-dependent cell death with DIDS and related compounds implicated ion transporters as important players during GAS infections, identifying possible protein targets for SLS in keratinocytes was complicated by the broad inhibitory activity of DIDS and other stilbene derivatives (Matulef and Maduke, 2005; Wulff, 2008; Boedtkjer et al., 2012; Chen et al., 2014). Therefore, we undertook a bioinformatics-based approach to identify plausible keratinocyte targets for SLS. We previously identified a putative binding region for SLS in Band 3 by using a synthetic peptide corresponding to amino acids 848-872 of Band 3 to inhibit SLS-dependent hemolysis (Higashi et al., 2016). Taking into account that this peptide likely inhibited hemolysis by competing with full-length, membrane-bound Band 3 for SLS binding, we used NCBI BLAST to identify other human proteins with similarity to the synthetic peptide sequence. This search largely identified members of the SLC4 family of bicarbonate transporters, which comprise ten genes encoding chloride/bicarbonate exchangers (including Band 3), a borate exchanger, and sodium-coupled bicarbonate transporters (Boron et al., 2009; Romero et al., 2013).

Bioinformatics analysis using this inhibitory peptide identified multiple members of the SLC4 protein family, of which Band 3 is a member (encoded by *SLC4A1*). Several of the proteins that exhibited the strongest E-value and percent identity were isoforms of the electroneutral sodium-bicarbonate cotransporter 1 (NBCn1, encoded by *SLC4A7*). These isoforms shared 66.67% identity with the query sequence, with an E-value of 2e-09 (data not shown), indicating that this region is shared across isoforms of NBCn1. Alignment of full-length Band 3 and NBCn1 indicated that the C-terminal region of Band 3 was highly similar to a C-terminal region of NBCn1, and this similarity was especially strong within the region spanned by the synthetic peptide (**Figure 1C**). NBCn1 is an integral membrane protein that is predominantly associated with the maintenance of intracellular pH (pH_i_) by moving bicarbonate across the cell membrane for the net effect of either acid loading or acid extrusion (Pushkin et al., 1999; Choi et al., 2000; Boron et al., 2009; Romero et al., 2013; Thornell and Bevensee, 2015). This function is related to that of Band 3, which is a chloride-bicarbonate exchanger that is also involved in ion exchange and maintenance of osmotic balance in erythrocytes (Jennings, 1989; Arakawa et al., 2015). Altogether, these results suggested that NBCn1 represents a plausible keratinocyte target for SLS during GAS infection.

### NBCn1 is expressed in HaCaT cells

Although our bioinformatics analysis indicated that NBCn1 was a candidate for targeting by SLS, it was unclear whether NBCn1 was expressed in our HaCaT cell line under our culture conditions. Previous results from the Human Protein Atlas had indicated that *SLC4A7* transcript was present in HaCaT cells, but validation of expression at the protein level in this cell line was lacking (Uhlén et al., 2015). Therefore, we isolated total RNA from HaCaT cells and performed RT-PCR using primers that spanned exon-exon junctions and corresponded to cDNA. RT-PCR generated a product of approximately 105 bp corresponding to *SLC4A7* mRNA, demonstrating that this gene was expressed in HaCaT cells under our culture conditions (**Figure S3A**). Expression of NBCn1 at the protein level was evaluated using Western blotting. HaCaT cells were lysed in a hypoosmotic lysis buffer, and membranes were isolated by ultracentrifugation. Probing these membrane samples and cytosolic fractions of cell lysates with an antibody to NBCn1 resulted in a band of around 30 kDa in the membrane fraction (**Figure S3B**). Full-length NBCn1 is around 136 kDa in size (UniProt Q9Y6M7). This size discrepancy could be explained by cleavage of the domain recognized by the antibody during the lysis process. Regardless, when taken together, these results demonstrated that the *SLC4A7* gene is expressed in HaCaT cells, along with a polypeptide corresponding to a portion of NBCn1. This further suggested that NBCn1 could be targeted by GAS *via* SLS during infection.

### Inhibition of sodium-bicarbonate cotransport limits SLS-dependent keratinocyte cytotoxicity

**Figure 2:**
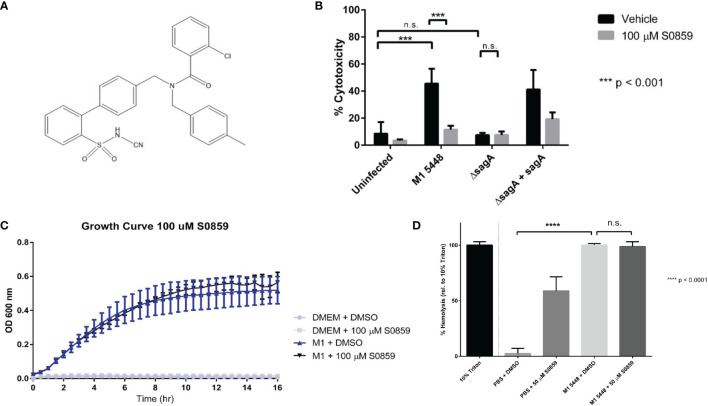
S0859 inhibits SLS-induced cytotoxicity in human keratinocytes, but not SLS-dependent hemolysis of erythrocytes. **(A)** Chemical structure of the N-cyanosulfonamide S0859. **(B)** Percent host cytotoxicity following 6 hour GAS infections of HaCaT cells (MOI 10) pre-treated for 1 hour with 100 µM S0859. Percent cytotoxicity was determined using an ethidium homodimer assay. DMSO was used as a vehicle control, and data are presented as mean ± sd, with n = 3 for each condition. **(C)** Growth curve of wild-type M1 5448 GAS in DMEM supplemented with 10% FBS in the presence or absence of 100 µM S0859. DMSO was used as a vehicle control, and un-inoculated DMEM + 10% FBS was used for each treatment condition as a sterility control. **(D)** Percent hemolysis (relative to 10% triton) induced by a 1 hour incubation of SLS preparations with erythrocytes in the presence of DMSO (Vehicle) or 50 µM S0859. *** = 0.0001 < P < 0.001, n.s. = not significant.

Our RT-PCR and Western blotting data suggested that NBCn1 was expressed in HaCaT cells (**Figure S3**). Therefore, we set out to determine whether this protein was targeted by SLS during GAS infection. Due to the broad inhibitory activity of DIDS and the stilbene derivatives discussed previously, we sought a more specific chemical inhibitor of sodium-bicarbonate cotransporters such as NBCn1 for use in GAS infections. Cells were treated with the N-cyanosulfonamide S0859 (whose structure is shown in **Figure 2A**), prior to infection with our panel of GAS strains. Unlike DIDS and the stilbene derivatives, S0859 is a much more specific inhibitor of sodium-bicarbonate cotransport, despite being unable to distinguish between individual sodium-bicarbonate cotransporters (Ch’en et al., 2008; Boedtkjer et al., 2012). When cells were treated with DMSO as a vehicle control, nearly 50 percent cytotoxicity was once again observed following infections with the SLS-producing GAS strains, but not in the uninfected or SLS-deficient *ΔsagA* controls (**Figure 2B**). Pre-treatment of keratinocytes with 100 μM S0859 resulted in a significant, SLS-dependent inhibition of cell death in our keratinocyte line, similar to what was seen with DIDS treatment (**Figure 2B**). In order to confirm that this inhibition was not due to S0859-dependent effects on GAS viability, we performed a growth curve analysis using wild-type GAS in the presence of DMSO or 100 µM S0859. This treatment had no apparent effect on GAS growth, which indicated that the inhibitory effects were due to S0859 treatment mitigating SLS-mediated pathology, and not the growth of the GAS strains in our infection conditions (**Figure 2C**). These results suggested that sodium-bicarbonate transport plays an important role during SLS-mediated cytotoxicity during GAS infections of HaCaT keratinocytes. We also evaluated whether S0859 treatment was capable of preventing SLS-mediated hemolysis in erythrocytes, and we determined that 50 µM S0859 treatment was unable to protect erythrocytes from hemolysis in response to SLS (**Figure 2D**). Interestingly, S0859 treatment alone induced hemolysis at an intermediate level between DMSO-treated cells and wild-type GAS-treated cells, suggesting that this compound induced an osmotic stress that the erythrocytes were partially sensitive to. However, there was no significant difference observed in hemolysis when erythrocytes were treated with DMSO or 50 µM S0859 in the presence of SLS preparations from wild-type GAS. Altogether, this indicated that S0859 treatment was able to prevent SLS-dependent effects in some cell types, but not others.

### S0859 treatment inhibits SLS-dependent NF-κB activation

In addition to causing cytotoxicity during HaCaT infection, SLS is also known to induce multiple cell signaling events prior to cell death. These include the activation of pro-inflammatory signals through the transcription factor NF-κB (Flaherty et al., 2015). Therefore, we hypothesized that S0859 treatment inhibited SLS-mediated NF-κB activation during GAS infections, preventing the inflammatory response and subsequent cytotoxicity. To test this, we performed GAS infections of S0859 pre-treated HaCaT cells on glass coverslips for 4 hours. This shorter time course was used to observe signaling events prior to the widespread increase in SLS-dependent cell death that occurred as the infection approached 6 hours. Following the infection, cells were fixed with paraformaldehyde and NF-κB p65 localization was assessed using immunofluorescence microscopy. NF-κB is a transcription factor that is typically bound to its inhibitor IκBα and localized to the cytoplasm. When activated by a stimulus, IκBα is degraded and NF-κB translocates to the cell nucleus, where it activates the transcription of pro-inflammatory genes (Liu et al., 2017). Therefore, the number of cells with NF-κB localized to the nucleus in a particular field can be used to assess the level of NF-κB activation in response to a stimulus, such as GAS infection.

**Figure 3:**
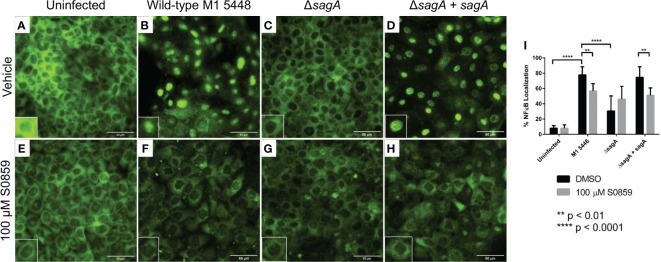
S0859 reduces SLS-dependent NF-κB activation in human keratinocytes. Representative immunofluorescence images (60x) of GAS infections after 4 hours (MOI 10) in the presence of vehicle (DMSO, **(A–D)** or 100 μM S0859 **(E–H)**. A Rb α NF-κB primary antibody (1:50) and Gt α Rb IgG AlexaFluor 488 (1:200) were used for this experiment. The scale bar indicates 50 µm. Minimal NF-κB nuclear localization was observed in the uninfected + vehicle **(A)**, uninfected + 100 μM S0859 **(E)**, ΔsagA + vehicle **(C)**, and ΔsagA + 100 μM S0859 **(G)** conditions. Most cells in the WT + vehicle **(B)** and ΔsagA + sagA + vehicle **(D)** exhibited strong nuclear localization of NF-κB, which was significantly reduced when these infections were performed in the presence of 100 μM S0859 **(F, H)**. Each panel has an inset that highlights a particular cell, indicating either NF-κB localized to the cytoplasm **(A, C, E–H)** or the nucleus **(B, D)**. **(I)** 10 fields from each condition were imaged, and the percentage of cells with NF-κB nuclear localization was calculated by counting the number of cells with NF-κB localized to the nucleus, and dividing by the total number of cells in the field.

When cells were pre-treated with the vehicle control DMSO, most cells showed NF-κB primarily in the cytoplasm in the uninfected control (**Figure 3A**) or when infected with the *ΔsagA* strain (**Figure 3C**). However, when HaCaT cells were infected with the SLS-producing wild-type (**Figure 3B**) and *ΔsagA + sagA* (**Figure 3D**) strains, the majority of cells experienced robust NF-κB localization to the cell nucleus. This aligned with previous results, indicating that SLS induced NF-κB activation in epithelial cells during GAS infection. When cells were pre-treated with S0859, minimal NF-κB activation was observed in the uninfected (**Figure 3E**) and *ΔsagA* (**Figure 3G**) conditions, similar to the DMSO-treated conditions. However, we also observed reduced NF-κB activation in S0859-treated cells infected with the wild-type (**Figure 3F**) and *ΔsagA + sagA* (**Figure 3H**) GAS strains. These effects were quantified by counting the number of cells with NF-κB localized to the nucleus relative to the total number of cells in 10 fields per condition, and this analysis demonstrated that treatment with S0859 significantly inhibited the ability of SLS to activate NF-κB (**Figure 3I**). Taken together, these results demonstrated that targeted inhibition of sodium-bicarbonate cotransport with S0859 reduces the ability of SLS to promote inflammation during GAS infection of epithelial cells.

### SLS causes intracellular acidification during GAS infection

NBCn1 and other SLC4 proteins that are involved in sodium-bicarbonate cotransport are often associated with net acid loading or extrusion, which is accomplished by moving bicarbonate ions across the cell membrane (Boron et al., 2009; Romero et al., 2013; Thornell and Bevensee, 2015). By extension, these proteins play a major role in the maintenance of internal pH (pH_i_). Given that chemical inhibition of sodium-bicarbonate cotransport with S0859 was able to reduce the pathological effects of SLS (**Figures 2**, **3**), we hypothesized that SLS would disrupt the ability of HaCaT cells to maintain their pH_i_ during infection. To test this, we loaded HaCaT cells with the pH-sensitive, fluorescent dye pHrodo Red AM, infected cells with live GAS, and monitored the fluorescent response within the cells. The high photostability and retention of pHrodo Red AM within cells makes ratiometric measurements unnecessary, and because the fluorescent intensity of pHrodo Red AM increases as pH_i_ decreases, a single fluorescence reading can be directly correlated to pH_i_ levels.

Therefore, we subjected pHrodo Red AM-loaded HaCaT cells to live GAS infections for 8 hours, and monitored fluorescence intensity over time using real-time live imaging. Infections were carried out at an MOI of 0.01 to prevent keratinocytes from being rapidly overrun by bacteria, and images were taken every 10 minutes over the course of the infection. Exposure time for the pHrodo channel was 10 ms across all conditions. Cells infected with either the wild-type or complemented *ΔsagA + sagA* strain of GAS experienced a sharp increase in fluorescence intensity over time, with some cell boundaries eventually becoming indistinguishable due to the strength of the fluorescence (**Videos S1A, S3A**). In contrast, cells infected with the SLS-deficient *ΔsagA* strain experienced a much more gradual increase in fluorescence intensity over time (**Video S2A**). This was clearly seen in frames taken every 120 minutes from representative videos for each infection condition, as shown in **Figure S4**. Images were also taken in the DIC channel to monitor bacterial growth over the course of the infection, and these videos indicated that bacterial growth was comparable between all conditions (**Videos S1B–S3B**). Eight cells per condition were tracked over the course of the 8 hours to observe changes in corrected total cell fluorescence over time, but the variability between cells within each condition resulted in large error that obscured any differences between the strains (data not shown). Regardless, our data demonstrated that infection of HaCaT cells with GAS strains that produce SLS experience a more dramatic intracellular acidification than those that are infected with GAS strains that do not produce SLS.

Having shown that SLS exposure resulted in a decrease in pH_i_, we hypothesized that S0859 exhibited its protective effects on keratinocyte cytotoxicity by preventing or disrupting SLS from inducing this intracellular acidification. To test this, we performed real-time live imaging experiments with HaCaT cells that were pre-treated with 100 µM S0859 prior to infection with wild-type GAS. Interestingly, these pre-treated cells also experienced a rapid increase in fluorescence intensity over the 8 hour infection period (**Video S4A**). The DIC channel indicated that bacterial growth was similar to what we observed in the videos of cells not treated with S0859 (**Video S4B**). These results indicated that SLS exposure in either the presence or absence of S0859 treatment caused a decrease in keratinocyte pH_i_, and suggested that S0859 inhibits SLS-mediated cytotoxicity through a mechanism that does not involve preventing an intracellular acidification event. Furthermore, it is likely that S0859 treatment alone would result in intracellular acidification by disrupting normal sodium-bicarbonate cotransport. Altogether, these results from monitoring pH_i_ changes demonstrated that epithelial cells experience strong intracellular acidification in response to SLS.

### S0859 treatment does not affect lesion size in a necrotizing model of GAS infection

**Figure 4:**
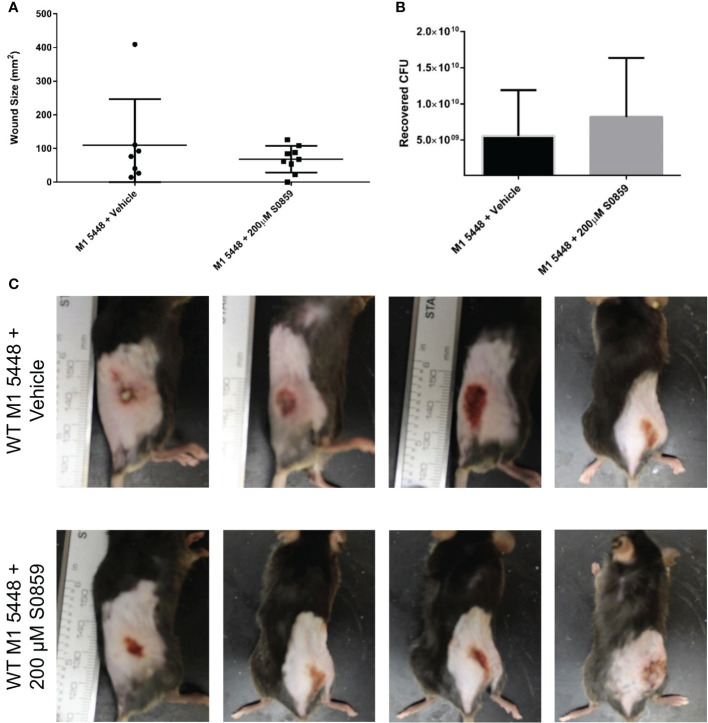
Multiple 200 µM S0859 treatments do not affect wound size in an *in vivo* model of GAS infection. C57BL/6 mice expressing the human plasminogen transgene were injected subcutaneously with wild-type M1 5448 GAS along with 0.7% saline (vehicle) or 200 µM S0859. Additional doses of saline or 200 µM S0859 were administered at 4 and 8 hours post-infection. After 24 hours, skin lesions were measured and imaged, and mice were sacrificed. Wounds were then excised and homogenized to determine GAS CFU. **(A)** Wound size in mm^2^ following a 24 hour infection. **(B)** Recovered CFU from the infected wounds after 24 hours. **(C)** Representative images of wounds from each group following the infection.

Having established that sodium-bicarbonate cotransport played a role in SLS-mediated GAS pathogenesis *in vitro*, we hypothesized that S0859 treatment would mitigate SLS-dependent effects *in vivo*. To test this, we used C57BL/6 mice expressing the human plasminogen transgene as a model system for GAS infection, which has been described previously (Sun et al., 2004; Higashi et al., 2016). Mice were injected with 10^7^ CFU of either wild-type (± 100 µM S0859), *ΔsagA*, or *ΔsagA + sagA* GAS, and were monitored for 3 days. Mice infected with wild-type GAS in both the presence and absence of S0859 treatment developed skin lesions within 24 hours of infection, while wounds in the *ΔsagA*, and *ΔsagA + sagA* conditions progressed more slowly (**Figure S5**). By 72 hours post-infection, there was no significant quantitative difference in wound size between wild-type-infected mice treated with the vehicle control or 100 µM S0859. S0859 has been described as a reversible inhibitor (Ch’en et al., 2008), so we hypothesized that additional injections of a higher dose of S0859 would be able to produce a treatment effect. We injected mice with wild-type GAS and either 200 µM S0859 or 0.7% saline as a vehicle control at the start of the infection, and then provided additional injections of either 200 µM S0859 or saline at 4 and 8 hours post-infection. Both groups of mice developed wounds within 24 hours, and there was no apparent difference in the size of these wounds (**Figures 4A, C**) or bacterial CFU recovered from the wounds between groups (**Figure 4B**). This indicated that S0859 was unable to inhibit SLS-mediated GAS virulence *in vivo*, which could be attributed to either the reversible nature of the inhibitor or its stability *in vivo*. In addition, the ability of SLS to still cause pathology in this model despite S0859 treatment indicates that SLS is still likely contributing to lesion development by exhibiting cytolytic effects on host cells other than keratinocytes, such as erythrocytes. This supports the hypothesis that SLS has multiple targets in different host cell types, because the *in vivo* system likely has multiple host cells and components that are targeted by SLS during GAS infection.

## Discussion

Here, we provide evidence that Streptolysin S-mediated cytotoxicity during GAS infection of human keratinocytes involves the disruption of pH homeostasis and the induction of host cell signaling events in response to the toxin. We demonstrate that cell death and pro-inflammatory NF-κB activation in response to SLS can be mitigated through chemical inhibition of proteins involved in ion transport across the cell membrane, such as NBCn1. Furthermore, our results show that treatment of keratinocytes with SLS results in intracellular acidification that does not occur in the absence of SLS or chemical inhibitors of sodium-bicarbonate cotransport. It has previously been reported that GAS infection results in activation of NF-κB and MAPK signaling in epithelial cells, and more recent studies have demonstrated that GAS causes these signaling events through SLS, leading to the production of pro-inflammatory cytokines such as IL-1β (Tsai et al., 2006; Flaherty et al., 2015; Flaherty et al., 2018). We now hypothesize that SLS produces these pro-inflammatory signaling cascades in the host by disrupting ion transporters involved in pH homeostasis in human keratinocytes during infection, eventually leading to host cytotoxicity. This is in conflict with the traditional view of SLS as a pore-forming toxin that nonspecifically disrupts membranes. However, the ability of SLS to target host proteins during infection has become increasingly well-known, and we have previously demonstrated that SLS induces hemolysis by targeting the SLC4 family member Band 3 in erythrocytes (Higashi et al., 2016).

Our results suggest that SLS targets host proteins involved in ion transport, and that treating keratinocytes with inhibitors of ion transporters prevents SLS-mediated effects *in vitro*. For example, treating keratinocytes with DIDS resulted in nearly complete inhibition of SLS-dependent cytotoxicity (**Figure 1B**). The ability of DIDS to inhibit NBCn1 appears to be highly variable and cell type dependent (Aalkjaer and Cragoe, 1988; Choi et al., 2000; Odgaard et al., 2004; Boedtkjer et al., 2012). Therefore, additional approaches were necessary to support the hypothesis that NBCn1 was a host target for SLS in keratinocytes. Pre-treatment of keratinocytes with S0859 was similarly able to inhibit SLS-dependent cytotoxicity (**Figure 2B**), as well as NF-κB activation in keratinocytes (**Figure 3**), without impacting bacterial growth (**Figure 2C**). S0859 has greater specificity for sodium-bicarbonate cotransporters such as NBCn1, despite being unable to distinguish between different sodium-bicarbonate cotransporters in the SLC4 family, and recent reports that it can inhibit the SLC16 family of monocarboxylate transporters (Ch’en et al., 2008; Lauritzen et al., 2010; Boedtkjer et al., 2012; Heidtmann et al., 2015). This suggests that proteins other than NBCn1, or combinations thereof, could be involved in the host response to SLS. Regardless, our results suggest that ion transporters play an important role in SLS-mediated pathogenesis in multiple cell types. The link between SLS and NBCn1 is further supported by the significant homology between a C-terminal region of NBCn1 and a C-terminal region of Band 3 that is a putative SLS-binding site (**Figure 1C**). This putative binding site corresponds to a synthetic peptide that, when incubated with SLS preparations and erythrocytes, was able to significantly inhibit SLS-dependent hemolysis (Higashi et al., 2016). Taken together, these results suggest that NBCn1 is a plausible keratinocyte target for SLS during GAS infection.

Several pathogens are known to express virulence factors that disrupt proteins involved in ion flux during infection. For example, *Salmonella enterica* Serovar Typhimurium produces a protein known as MgtC that was shown to constitutively activate the host Na^+^, K^+^-ATPase, although more recent studies have questioned the physiological relevance of this interaction (Günzel et al., 2006; Lee et al., 2013). *Pseudomonas aeruginosa* uses multiple factors to reduce epithelial ion transport during lung infections, including the epoxide hydrolase CFTR inhibitory factor (Cif) that depletes host CFTR levels, and the alkaline protease AprA, which over-activates the epithelial sodium channel ENaC (Bomberger et al., 2011; Butterworth et al., 2012; Ballok and O’Toole, 2013). The disruption of various ion channels is also a major contributor to enteric diseases caused by a variety of pathogens. Ion transporters implicated in these diseases include the sodium-hydrogen exchangers NHE2 and NHE3, which are disrupted by cholera toxin, and NHE3 activity is also reduced by TcdB produced by *Clostridium dificile* (Hayashi et al., 2004; Subramanya et al., 2007; Engevik et al., 2015; Das et al., 2018). Multiple pathogens also target ion transporters in order to evade the host immune system (Westman and Grinstein, 2021). One such example is *Legionella pneumophilia*, which uses the effector SidK to bind and inhibit a proton-pumping vacuolar-type ATPase, enabling the bacteria to prevent acidification of the phagolysosome and promote intracellular survival (Zhao et al., 2017). Altogether, these studies highlight the importance of ion transporters in the host to a variety of mechanisms that are involved in bacterial virulence, and our results identify an additional ion transporter that is disrupted by a pathogen to contribute to disease progression.

NBCn1 and other sodium-bicarbonate cotransporters often contribute to the regulation of cytoplasmic pH in various cell types (Boron et al., 2009; Romero et al., 2013). Our results suggest that SLS induces intracellular acidification in epithelial cells (**Figure S4**), which is consistent with this information. However, S0859 treatment does not prevent the acidification of cells infected with wild-type GAS (**Video S4**), despite protecting keratinocytes from SLS-mediated NF-κB activation (**Figure 3**) and cytotoxicity (**Figure 2B**). This is in agreement with the association of NBCn1 with acid extrusion, and S0859 has previously been shown to prevent sodium-bicarbonate cotransport-mediated pH_i_ recovery in response to an acidification event (Leem et al., 1999; Ch’en et al., 2008). The ability of host cells to tightly regulate their intracellular pH is crucial for their capability to control pathogens during infection. One manifestation of this is the process of phagocytosis, which occurs in both innate immune cells and epithelial cells (Sedlyarov et al., 2018; Sharma et al., 2020). This process must be rapid enough to clear bacterial components quickly to prevent further infection, but also must be controlled to avoid damaging hyper-acidification (Coakley et al., 2002; Sedlyarov et al., 2018). Therefore, the host has numerous mechanisms in place to regulate pH_i_, many of which involve acid loading or extrusion through ion transporters, channels, or proton pumps (Boron, 1986). The solute carrier 4 (SLC4) family of ion exchangers comprise ten members, nine of which are involved in bicarbonate flux across the cell membrane (Boron et al., 2009; Romero et al., 2013). In particular, NBCn1 (encoded by *SLC4A7*) is known to be strongly induced during macrophage differentiation and play a role in phagolysosome acidification, suggesting that this transporter plays a role in host responses to pathogens (Sedlyarov et al., 2018).

We hypothesize that when GAS infects human keratinocytes, bacteria are phagocytosed, and keratinocytes must properly regulate their pH_i_ to process bacterial components. In the absence of S0859, SLS binds and disrupts NBCn1, resulting in improper regulation of intracellular acidification. This induces a cellular stress response, activating NF-κB expression and the production of pro-inflammatory cytokines, leading to cytotoxicity. S0859 is able to exert its protective effect by inhibiting NBCn1, preventing SLS from disrupting the transporter and allowing the host cell to acidify more rapidly to degrade the bacterial components. However, we cannot rule out the possibility that SLS targeting NBCn1 induces inflammation without directly impacting pH_i_. In this case, exposure to SLS would result in an over-activated inflammatory response, and eventually intracellular acidification and cell death, which could be inhibited by S0859. This possibility is less likely, due to the ability of S0859 to also inhibit the SLS-induced activation of NF-κB (**Figure 3**). Regardless, there is a strong link between pH regulation, inflammation, and cell viability (Hackam et al., 1996; Coakley et al., 2002; Rajamäki et al., 2013; Murase et al., 2018; Yambire et al., 2019; Wong et al., 2021), and our results suggest that SLS is able to disrupt this link to promote cell death.

In order to explore the role of sodium-bicarbonate cotransport in SLS-mediated GAS virulence *in vivo*, we used a humanized mouse model for GAS infection in the presence of S0859 treatment. Although we have not evaluated the ability of S0859 to inhibit SLS-mediated effects in murine epithelial cells, this compound is a well-characterized generic inhibitor of sodium-bicarbonate cotransport that can inhibit proteins involved in this process in cells from multiple species, including mice, rats, guinea pigs, rabbits, and humans (Ch’en et al., 2008; Dagnell et al., 2019; Kawakami et al., 2020). Therefore, we expected that S0859 treatment would inhibit sodium-bicarbonate cotransport in our mouse model, allowing us to observe the effects of SLS-mediated GAS pathology. Surprisingly, the size of necrotic wounds formed by SLS-producing GAS was not affected by treatment with 100 µM S0859 at the start of the infection (**Figure S5**) or with 200 µM S0859 at the start of infection followed by additional doses at 4 and 8 hours post-infection (**Figure 4**). This does not reflect the strong inhibitory effects of S0859 that we observed *in vitro*, indicating that GAS pathogenesis is a complex process that involves multiple interactions between factors produced by both the host and pathogen. Previously, the stilbenedisulfonate DIDS has been shown to cause a reduction in wound size as a response to SLS in this model, indicating that the progression of GAS pathology can be disrupted by pharmacologically targeting SLS (Higashi et al., 2016). Therefore, DIDS can inhibit SLS-mediated cytotoxicity in human keratinocytes (**Figure 1B**), SLS-mediated hemolysis in erythrocytes (Higashi et al., 2016), and the formation of SLS-dependent skin lesions in mice, while S0859 can only inhibit keratinocyte cell death (**Figures 2**, **4**). This could be explained by covalent and irreversible binding of DIDS to its proteins targets (Okubo et al., 1994; Boedtkjer et al., 2012), compared to the reversible nature of binding between S0859 and sodium-bicarbonate cotransporters (Ch’en et al., 2008), which would suggest that concentrations of S0859 at the site of infection may not remain sufficiently high to prevent SLS from disrupting NBCn1. However, we attribute this variation to the range of proteins that can be inhibited by these compounds. The ability of DIDS to inhibit a variety of proteins would allow this compound to prevent SLS from targeting host proteins in multiple cell types simultaneously, while the more specific inhibitory range of S0859 suggests that it would only be able to prevent SLS from interacting with the NBCn1 on keratinocytes, but not other targets such as Band 3 in erythrocytes. This information underscores the importance of understanding bacterial effectors that have multiple host targets, since chemical treatments that inhibit a particular toxin in a particular *in vitro* system may not be effective *in vivo* due to additional targets being available, allowing the bacterial effector to exert its activity through alternative mechanisms. Therefore, we suggest that host-pathogen studies involving the inhibition of bacterial toxins should consider the use of multiple cell models to allow for a better understanding of how toxins affect the host during infection, and more precision when developing therapeutic approaches in these systems.

In summary, we have demonstrated that ion transporters play an important role in the response of the host to the Group A *Streptococcal* virulence factor Streptolysin S. Our results show that GAS uses SLS to induce signaling events and promote programmed cell death in human keratinocytes by targeting the sodium-bicarbonate cotransporter NBCn1. These effects can be prevented by treating keratinocytes with the chemical inhibitor S0859, although this compound does not exhibit inhibitory effects in an *in vivo* model. Regardless, our *in vitro* results suggest that designing a chemical library around S0859 holds promise for the development of therapeutics for the treatment of severe GAS infections. Furthermore, we show that SLS disrupts the ability of host cells to regulate their internal pH, suggesting a possible link between bacterial pathogenesis, pH homeostasis, and host cell responses to infection. Taken together, our results support the idea that GAS uses SLS as a multifunctional weapon to manipulate its environment in multiple context-dependent ways, allowing the pathogen to promote severe disease during infection. These results have important implications for understanding how bacteria produce effectors to disrupt physiological processes in the host for their benefit, allowing them to be more effective pathogens by co-opting existing host processes during infections.

## Data availability statement

The datasets presented in this study can be found in online repositories. The names of the repository/repositories and accession number(s) can be found in the article/**Supplementary Material**.

## Ethics statement

The animal study was reviewed and approved by University of Notre DAME IACUC.

## Author contributions

DH and SL designed the project and experimental aims, and wrote the manuscript. DH, DD, and ZT performed experimental work and analyzed the results. All authors contributed to the proofreading and editing of the manuscript. All authors contributed to the article and approved the submitted version.

## Funding

DH was supported by the Arthur J. Schmitt Presidential Leadership Fellowship. The study was funded by a National Institutes of Health Grant (R01 HL13423) to FC, VP, SL.

## Conflict of interest

The authors declare that the research was conducted in the absence of any commercial or financial relationships that could be construed as a potential conflict of interest.

## Publisher’s note

All claims expressed in this article are solely those of the authors and do not necessarily represent those of their affiliated organizations, or those of the publisher, the editors and the reviewers. Any product that may be evaluated in this article, or claim that may be made by its manufacturer, is not guaranteed or endorsed by the publisher.

## References

[B1] Aalkjaer C. Cragoe E. J. (1988). Intracellular pH regulation in resting and contracting segments of rat mesenteric resistance vessels. J. Physiol. 402, 391. doi: 10.1113/JPHYSIOL.1988.SP017211 2976824 PMC1191898

[B2] Altschul S. F. Madden T. L. Schäffer A. A. Zhang J. Zhang Z. Miller W.. (1997). Gapped BLAST and PSI-BLAST: A new generation of protein database search programs. Nucleic Acids Res. 25, 3389–3402. doi: 10.1093/nar/25.17.3389 9254694 PMC146917

[B3] Arakawa T. Kobayashi-Yurugi T. Alguel Y. Iwanari H. Hatae H. Iwata M.. (2015). Crystal structure of the anion exchanger domain of human erythrocyte band 3. Science 80) 350, 680–684. doi: 10.1126/science.aaa4335 26542571

[B4] Ballok A. E. O’Toole G. A. (2013). Pouring salt on a wound: Pseudomonas aeruginosa virulence factors alter na+ and cl- flux in the lung. J. Bacteriol. 195, 4013–4019. doi: 10.1128/JB.00339-13 23836869 PMC3754746

[B5] Barnett T. C. Cole J. N. Rivera-Hernandez T. Henningham A. Paton J. C. Nizet V.. (2015). Streptococcal toxins: Role in pathogenesis and disease. Cell. Microbiol. 17, 1721–1741. doi: 10.1111/cmi.12531 26433203

[B6] Berube B. J. Bubeck Wardenburg J. (2013). Staphylococcus aureus α-toxin: nearly a century of intrigue. Toxins (Basel). 5, 1140–1166. doi: 10.3390/TOXINS5061140 23888516 PMC3717774

[B7] Betschel S. D. Borgia S. M. Barg N. L. Low D. E. De Azavedo J. C. S. (1998). Reduced virulence of group a streptococcal Tn916 mutants that do not produce streptolysin s. Infect. Immun. 66, 1671–1679. doi: 10.1128/IAI.66.4.1671-1679.1998 9529097 PMC108104

[B8] Boedtkjer E. Bunch L. Pedersen S. F. (2012). Physiology, pharmacology and pathophysiology of the pH regulatory transport proteins NHE1 and NBCn1: Similarities, differences, and implications for cancer therapy. Curr. Pharm. Des. 18, 1345–1371. doi: 10.2174/138161212799504830 22360557

[B9] Bomberger J. M. Ye S. MacEachran D. P. Koeppen K. Barnaby R. L. O’Toole G. A.. (2011). A pseudomonas aeruginosa toxin that hijacks the host ubiquitin proteolytic system. PloS Pathog. 7, e1001325. doi: 10.1371/JOURNAL.PPAT.1001325 21455491 PMC3063759

[B10] Boron W. F. (1986). Intracellular pH regulation in epithelial cells. Annu. Rev. Physiol. 48, 377–388. doi: 10.1146/ANNUREV.PH.48.030186.002113 3010818

[B11] Boron W. F. Chen L. Parker M. D. (2009). Modular structure of sodium-coupled bicarbonate transporters. J. Exp. Biol. 212, 1697–1706. doi: 10.1242/jeb.028563 19448079 PMC2683013

[B12] Boukamp P. Petrussevska R. T. Breitkreutz D. Hornung J. Markham A. Fusenig N. E. (1988). Normal keratinization in a spontaneously immortalized aneuploid human keratinocyte cell line. J. Cell Biol. 106, 761–771. doi: 10.1083/jcb.106.3.761 2450098 PMC2115116

[B13] Butterworth M. B. Zhang L. Heidrich E. M. Myerburg M. M. Thibodeau P. H. (2012). Activation of the epithelial sodium channel (ENaC) by the alkaline protease from pseudomonas aeruginosa. J. Biol. Chem. 287, 32556–32565. doi: 10.1074/JBC.M112.369520 22859302 PMC3463336

[B14] Carapetis J. R. Steer A. C. Mulholland E. K. Weber M. (2005). The global burden of group a streptococcal diseases. Lancet Infect. Dis. 5, 685–694. doi: 10.1016/S1473-3099(05)70267-X 16253886

[B15] Castiglia V. Piersigilli A. Ebner F. Janos M. Goldmann O. Damböck U.. (2016). Type i interferon signaling prevents IL-1β-Driven lethal systemic hyperinflammation during invasive bacterial infection of soft tissue. Cell Host Microbe 19, 375–387. doi: 10.1016/j.chom.2016.02.003 26962946

[B16] Chen H. Gao W. Yang Y. Guo S. Wang H. Wang W.. (2014). Inhibition of VDAC1 prevents Ca2+-mediated oxidative stress and apoptosis induced by 5-aminolevulinic acid mediated sonodynamic therapy in THP-1 macrophages. Apoptosis 19, 1712–1726. doi: 10.1007/s10495-014-1045-5 25342393

[B17] Ch’en F. Villafuerte F. C. Swietach P. Cobden P. M. Vaughan-jones R. D. (2008). S0859, an n-cyanosulphonamide inhibitor of sodium-bicarbonate cotransport in the heart. Br. J. Pharmacol. 153, 972–982. doi: 10.1038/sj.bjp.0707667 18204485 PMC2267275

[B18] Choi I. Aalkjaer C. Boulpaep E. L. (2000). An electroneutral sodium / bicarbonate cotransporter NBCn1 and associated sodium channel. Nature 405, 571–575. doi: 10.1038/35014615 10850716

[B19] Coakley R. J. Taggart C. McElvaney N. G. O’Neill S. J. (2002). Cytosolic pH and the inflammatory microenvironment modulate cell death in human neutrophils after phagocytosis. Blood 100, 3383–3391. doi: 10.1182/blood.V100.9.3383 12384441

[B20] Cotter P. D. Draper L. A. Lawton E. M. Daly K. M. Groeger D. S. Casey P. G.. (2008). Listeriolysin s, a novel peptide haemolysin associated with a subset of lineage I listeria monocytogenes. PloS Pathog. 4, 1–10. doi: 10.1371/journal.ppat.1000144 PMC2522273 18787690

[B21] Cunningham M. W. (2000). Pathogenesis of group a streptococcal infections. Clin. Microbiol. Rev. 13, 470–511. doi: 10.1128/CMR.13.3.470-511.2000 10885988 PMC88944

[B22] Dagnell M. Cheng Q. Rizvi S. H. M. Pace P. E. Boivin B. Winterbourn C. C.. (2019). Bicarbonate is essential for protein-tyrosine phosphatase 1B (PTP1B) oxidation and cellular signaling through EGF-triggered phosphorylation cascades. J. Biol. Chem. 294, 12330–12338. doi: 10.1074/jbc.RA119.009001 31197039 PMC6699834

[B23] Das S. Jayaratne R. Barrett K. E. (2018). The role of ion transporters in the pathophysiology of infectious diarrhea. Cell. Mol. Gastroenterol. Hepatol. 6, 33. doi: 10.1016/J.JCMGH.2018.02.009 29928670 PMC6007821

[B24] Datta V. Myskowski S. M. Kwinn L. A. Chiem D. N. Varki N. Kansal R. G.. (2005). Mutational analysis of the group a streptococcal operon encoding streptolysin s and its virulence role in invasive infection. Mol. Microbiol. 56, 681–695. doi: 10.1111/j.1365-2958.2005.04583.x 15819624

[B25] Duncan J. L. Mason L. (1976). Characteristics of streptolysin s hemolysis. Infect. Immun. 14, 77–82. doi: 10.1128/iai.14.1.77-82.1976 985807 PMC420846

[B26] Engevik M. A. Engevik K. A. Yacyshyn M. B. Wang J. Hassett D. J. Darien B.. (2015). Human clostridium difficile infection: inhibition of NHE3 and microbiota profile. Am. J. Physiol. Gastrointest. Liver Physiol. 308, G497–G509. doi: 10.1152/AJPGI.00090.2014 25552580 PMC4422371

[B27] Flaherty R. A. Donahue D. L. Carothers K. E. Ross J. N. Henry T. (2018). Neutralization of streptolysin s-dependent and independent inflammatory cytokine IL-1β activity reduces pathology during early group a streptococcal skin infection. Front. Cell. Infect. Microbiol. 8. doi: 10.3389/fcimb.2018.00211 PMC6037840 30018884

[B28] Flaherty R. A. Puricelli J. M. Higashi D. L. Park C. J. Lee S. W. (2015). Streptolysin s promotes programmed cell death and enhances inflammatory signaling in epithelial keratinocytes during group a streptococcus infection. Infect. Immun. 83, 4118–4133. doi: 10.1128/IAI.00611-15 26238711 PMC4567650

[B29] Galdiero S. Gouaux E. (2004). High resolution crystallographic studies of α-hemolysin–phospholipid complexes define heptamer–lipid head group interactions: Implication for understanding protein–lipid interactions. Protein Sci. 13, 1503. doi: 10.1110/PS.03561104 15152085 PMC2279993

[B30] Ginsburg I. (1972). Mechanisms of cell and tissue injury induced by group a Streptococci : Relation to poststreptococcal sequelae. Oxford Univ. Press 126, 294–340. doi: 10.1093/infdis/126.4.419 4627328

[B31] Günzel D. Kucharski L. M. Kehres D. G. Romero M. F. Maguire M. E. (2006). The MgtC virulence factor of salmonella enterica serovar typhimurium activates Na+,K+-ATPase. J. Bacteriol. 188, 5586–5594. doi: 10.1128/JB.00296-06/FORMAT/EPUB 16855249 PMC1540036

[B32] Hackam D. J. Grinstein S. Rotstein O. D. (1996). Intracellular pH regulation in leukocytes: Mechanisms and functional significance. Shock 5 (1), 17–21. doi: 10.1097/00024382-199601000-00005 8821098

[B33] Hayashi H. Szászi K. Coady-Osberg N. Furuya W. Bretscher A. P. Orlowski J.. (2004). Inhibition and redistribution of NHE3, the apical Na+/H+ exchanger, by clostridium difficile toxin b. J. Gen. Physiol. 123, 491–504. doi: 10.1085/JGP.200308979 15078917 PMC2234495

[B34] Heddle J. G. Blance S. J. Zamble D. B. Hollfelder F. Miller D. A. Wentzell L. M.. (2001). The antibiotic microcin B17 is a DNA gyrase poison: Characterisation of the mode of inhibition. J. Mol. Biol. 307, 1223–1234. doi: 10.1006/jmbi.2001.4562 11292337

[B35] Heidtmann H. Ruminot I. Becker H. M. Deitmer J. W. (2015). Inhibition of monocarboxylate transporter by n-cyanosulphonamide S0859. Eur. J. Pharmacol. 762, 344–349. doi: 10.1016/j.ejphar.2015.05.049 26027796

[B36] Henningham A. Barnett T. C. Maamary P. G. Walker M. J. (2012). Pathogenesis of group a streptococcal infections. Discovery Med. 13, 329–342. doi: 10.1128/cmr.13.3.470 22642914

[B37] Higashi D. L. Biais N. Donahue D. L. Mayfield J. A. Tessier C. R. Rodriguez K.. (2016). Activation of band 3 mediates group a streptococcus streptolysin s-based beta-haemolysis. Nat. Microbiol. 1, 1–6. doi: 10.1038/nmicrobiol.2015.4 27571972

[B38] Hirose Y. Yamaguchi M. Okuzaki D. Motooka D. Hamamoto H. Hanada T. (2019). Streptococcus pyogenes transcriptome changes in the inflammatory environment of necrotizing fasciitis. Appl. Environ. Microbiol. 85, 1–17. doi: 10.1128/AEM.01428-19 PMC6803311 31471300

[B39] Howery A. E. Elvington S. Abraham S. J. Choi K. Dworschak-simpson S. Phillips S.. (2012). A designed inhibitor of a CLC antiporter blocks function through a unique binding mode. Chem. Biol. 19, 1460–1470. doi: 10.1016/j.chembiol.2012.09.017 23177200 PMC3508466

[B40] Jennings M. L. (1989). Structure and function of the red blood cell anion transport protein Annu. Rev. Biophys. Biophys. Chem. 18, 397–430. doi: 10.1007/978-1-4612-4500-1_8 2660831

[B41] Kansal R. G. McGeer A. Low D. E. Norrby-Teglund A. Kotb M. (2000). Inverse relation between disease severity and expression of the streptococcal cysteine protease, SpeB, among clonal M1T1 isolates recovered from invasive group a streptococcal infection cases. Infect. Immun. 68, 6362–6369. doi: 10.1128/IAI.68.11.6362-6369.2000 11035746 PMC97720

[B42] Kawakami T. Koike A. Maehara T. Hayashi T. Fujimori K. (2020). Bicarbonate enhances the inflammatory response by activating JAK/STAT signalling in LPS 1 IFN-c-stimulated macrophages. J. Biochem. 167, 623–631. doi: 10.1093/jb/mvaa010 31960927

[B43] Lauritzen G. Jensen M. B. F. Boedtkjer E. Dybboe R. Aalkjaer C. Nylandsted J.. (2010). NBCn1 and NHE1 expression and activity in ΔNErbB2 receptor-expressing MCF-7 breast cancer cells: Contributions to pHi regulation and chemotherapy resistance. Exp. Cell Res. 316, 2538–2553. doi: 10.1016/j.yexcr.2010.06.005 20542029

[B44] Lee S. W. Mitchell D. A. Markley A. L. Hensler M. E. Gonzalez D. Wohlrab A.. (2008). Discovery of a widely distributed toxin biosynthetic gene cluster. Proc. Natl. Acad. Sci. U. S. A. 105, 5879–5884. doi: 10.1073/pnas.0801338105 18375757 PMC2311365

[B45] Leem C. H. Lagadic-Gossmann D. Vaughan-Jones R. D. (1999). Characterization of intracellular pH regulation in the guinea-pig ventricular myocyte. J. Physiol. 517, 159. doi: 10.1111/J.1469-7793.1999.0159Z.X 10226157 PMC2269328

[B46] Lee E. J. Pontes M. H. Groisman E. A. (2013). A bacterial virulence protein promotes pathogenicity by inhibiting the bacterium’s own F1Fo ATP synthase. Cell 154, 146. doi: 10.1016/J.CELL.2013.06.004 23827679 PMC3736803

[B47] Lei B. Minor D. Feng W. Jerome M. Quinn M. T. Jutila M. A. (2019). Tissue tropism in streptococcal infection: Wild-type M1T1 group a streptococcus is efficiently cleared by neutrophils using an NADPH oxidase-dependent mechanism in the lung but not in the skin. Infect. Immun. 87, 1–17. doi: 10.1128/IAI.00527-19 PMC6759303 31331954

[B48] Liu M. Lei B. (2018). Pathogenesis of hypervirulent group a streptococcus. Jpn J. Med. 1, 269–275. doi: 10.31488/jjm.1000127.Pathogenesis PMC6294136 30556051

[B49] Liu T. Zhang L. Joo D. Sun S. C. (2017). NF-κB signaling in inflammation. Signal Transduction Targeting Ther. 2, 1–9. doi: 10.1038/sigtrans.2017.23 PMC5661633 29158945

[B50] Matulef K. Howery A. E. Tan L. Kobertz W. R. Bois J. Maduke M. (2008). Discovery of potent CLC chloride channel inhibitors. ACS Chem. Biol. 3, 419–428. doi: 10.1021/cb800083a 18642799 PMC2556891

[B51] Matulef K. Maduke M. (2005). Side-dependent inhibition of a prokaryotic ClC by DIDS. Biophys. J. 89, 1721–1730. doi: 10.1529/biophysj.105.066522 15994902 PMC1366676

[B52] Mitchell D. A. Lee S. W. Pence M. A. Markley A. L. Limm J. D. Nizet V.. (2009). Structural and functional dissection of the heterocyclic peptide cytotoxin streptolysin s. J. Biol. Chem. 284, 13004–13012. doi: 10.1074/jbc.M900802200 19286651 PMC2676033

[B53] Miyoshi-Akiyama T. Takamatsu D. Koyanagi M. Zhao J. Imanishi K. Uchiyama T. (2005). Cytocidal effect of streptococcus pyogenes on mouse neutrophils *In vivo* and the critical role of streptolysin s. J. Infect. Dis. 192, 107–116. doi: 10.1086/430617 15942900

[B54] Molloy E. M. Cotter P. D. Hill C. Mitchell D. A. Ross R. P. (2014). Streptolysin s-like virulence factors: The continuing sagA. Nat. Rev. Microbiol. 9, 670–681. doi: 10.1038/nrmicro2624 PMC3928602 21822292

[B55] Murase M. Kawasaki T. Hakozaki R. Sueyoshi T. Putri D. D. P. Kitai Y.. (2018). Intravesicular acidification regulates lipopolysaccharide inflammation and tolerance through TLR4 trafficking. J. Immunol. 200, 2798–2808. doi: 10.4049/JIMMUNOL.1701390 29540576

[B56] Nizet V. Beall B. Bast D. J. Datta V. Kilburn L. Low D. E.. (2000). Genetic locus for streptolysin s production by group a streptococcus. Infect. Immun. 68, 4245–4254. doi: 10.1128/IAI.68.7.4245-4254.2000 10858242 PMC101736

[B57] Odgaard E. Jakobsen J. K. Frische S. Praetorius J. Nielsen S. Aalkjær C.. (2004). Basolateral na+-dependent HCO3- transporter NBCn1-mediated HCO3- influx in rat medullary thick ascending limb. J. Physiol. 555, 205–218. doi: 10.1113/JPHYSIOL.2003.046474 14673192 PMC1664813

[B58] Okazaki H. (1971). Streptolysin s of streptococcus activity pyogenes: Studies on phospholipase activity. J. Biochem. 70, 867–868. doi: 10.1093/oxfordjournals.jbchem.a129704 4947362

[B59] Okubo K. Kang D. Hamasaki N. Jennings M. (1994). Lysine 539 and lysine 851 react with the same H2DIDS (4,4’-diisothiocyanodihydrostilbene-2,2’-disulfonic acid) molecule. J. Biol. Chem. 269, 1918–1926. doi: 10.1016/S0021-9258(17)42114-4 8294441

[B60] Powers M. E. Kim H. K. Wang Y. Wardenburg J. B. (2012). ADAM10 mediates vascular injury induced by staphylococcus aureus α-hemolysin. J. Infect. Dis. 206, 352–356. doi: 10.1093/INFDIS/JIS192 22474035 PMC3392186

[B61] Pushkin A. Abuladze N. Lee I. Newman D. Hwang J. Kurtz I. (1999). Cloning, tissue distribution, genomic organization, and functional characterization of NBC3, a new member of the sodium bicarbonate cotransporter family. J. Biol. Chem. 274, 16569–16575. doi: 10.1074/jbc.274.23.16569 10347222

[B62] Rajamäki K. Nordström T. Nurmi K. Åkerman K. E. O. Kovanen P. T. Öörni K.. (2013). Extracellular acidosis is a novel danger signal alerting innate immunity *via* the NLRP3 inflammasome. J. Biol. Chem. 288, 13410. doi: 10.1074/JBC.M112.426254 23530046 PMC3650379

[B63] Romero M. F. Chen A. P. Parker M. D. Boron W. F. (2013). The SLC4 family of bicarbonate (HCO3-) transporters. Mol. Aspects Med. 34, 159–182. doi: 10.1016/j.mam.2012.10.008 23506864 PMC3605756

[B64] Roy R. S. Gehring A. M. Milne J. C. Belshaw P. J. Christopher T. (1999). Thiazole and oxazole peptides: biosynthesis and molecular machinery. Nat. Prod. Rep. 16, 249–263. doi: 10.1039/a806930a 10331285

[B65] Sedlyarov V. Eichner R. Girardi E. Kovarik P. Demaurex N. Superti-furga G.. (2018). The bicarbonate transporter SLC4A7 plays a key role in macrophage phagosome acidification. Cell Host Microbe 23, 766–774. doi: 10.1016/j.chom.2018.04.013 29779931 PMC6002608

[B66] Sharma L. Feng J. Britto C. J. Dela Cruz C. S. (2020). Mechanisms of epithelial immunity evasion by respiratory bacterial pathogens. Front. Immunol. 11. doi: 10.3389/FIMMU.2020.00091/BIBTEX PMC7027138 32117248

[B67] Sievers F. Wilm A. Dineen D. Gibson T. J. Karplus K. Li W.. (2011). Fast, scalable generation of high-quality protein multiple sequence alignments using clustal omega. Mol. Syst. Biol. 7, 1–6. doi: 10.1038/msb.2011.75 PMC3261699 21988835

[B68] Subramanya S. B. Rajendran V. M. Srinivasan P. Nanda Kumar N. S. Ramakrishna B. S. Binder H. J. (2007). Differential regulation of cholera toxin-inhibited Na-h exchange isoforms by butyrate in rat ileum. Am. J. Physiol. Gastrointest. Liver Physiol. 293, 857–863. doi: 10.1152/AJPGI.00462.2006 17690171

[B69] Sumitomo T. Nakata M. Higashino M. Jin Y. Terao Y. Fujinaga Y.. (2011). Streptolysin s contributes to group a streptococcal translocation across an epithelial barrier. J. Biol. Chem. 286, 2750–2761. doi: 10.1074/jbc.M110.171504 21084306 PMC3024771

[B70] Sun H. Ringdahl U. Homeister J. W. Fay W. P. Engleberg N. C. Yang A. Y.. (2004). Plasminogen is a critical host pathogenicity factor for group a streptococcal infection. Science 80-. ). doi: 10.1126/science.1101245 15333838

[B71] Thornell I. M. Bevensee M. O. (2015). Regulators of Slc4 bicarbonate transporter activity. Front. Physiol. 6. doi: 10.3389/fphys.2015.00166 PMC4464172 26124722

[B72] Todd E. W. (1938). The differentiation of two distinct serological varieties of streptolysin, streptolysin O and streptolysin s. J. Pathol. 47, 423–445. doi: 10.1002/path.1700470307

[B73] Tsai P. J. Chen Y. H. Hsueh C. H. Hsieh H. C. Liu Y. H. Wu J. J.. (2006). Streptococcus pyogenes induces epithelial inflammatory responses through NF-κB/MAPK signaling pathways. Microbes Infect. 8, 1440–1449. doi: 10.1016/j.micinf.2006.01.002 16702013

[B74] Uhlén M. Fagerberg L. Hallström B. M. Lindskog C. Oksvold P. Mardinoglu A.. (2015). Tissue-based map of the human proteome. Science 80, 1–9. doi: 10.1126/science.1260419 25613900

[B75] Vizan J. L. Hernandez-chico C. Castillo I. Moreno F. (1991). The peptide antibiotic microcin bi 7 induces double-strand cleavage of DNA mediated by e. coli 10, 467–476. doi: 10.1002/j.1460-2075.1991.tb07969.x PMC452668 1846808

[B76] von Beek C. Waern I. Eriksson J. Melo F. R. Robinson C. Waller A. S.. (2019). Streptococcal sagA activates a proinflammatory response in mast cells by a sublytic mechanism. Cell. Microbiol. 21, 1–15. doi: 10.1111/cmi.13064 PMC6771685 31155820

[B77] Waddington C. S. Snelling T. L. Carapetis J. R. (2014). Management of invasive group a streptococcal infections. J. Infect. 69, S63–S69. doi: 10.1016/j.jinf.2014.08.005 25307276

[B78] Walker M. J. Barnett T. C. McArthur J. D. Cole J. N. Gillen C. M. Henningham A.. (2014). Disease manifestations and pathogenic mechanisms of group a streptococcus. Clin. Microbiol. Rev. 27, 264–301. doi: 10.1128/CMR.00101-13 24696436 PMC3993104

[B79] Westman J. Grinstein S. (2021). Determinants of phagosomal pH during host-pathogen interactions. Front. Cell Dev. Biol. 8. doi: 10.3389/FCELL.2020.624958/BIBTEX PMC7829662 33505976

[B80] Wilke G. A. Wardenburg J. B. (2010). Role of a disintegrin and metalloprotease 10 in staphylococcus aureus α-hemolysin - mediated cellular injury. Proc. Natl. Acad. Sci. U.S.A. 107, 13473–13478. doi: 10.1073/PNAS.1001815107/SUPPL_FILE/PNAS.201001815SI.PDF 20624979 PMC2922128

[B81] Wong C. W. Pratiwi F. W. Chen P. Mou C. Y. Hsu S. H. (2021). Revealing the phagosomal pH regulation and inflammation of macrophages after endocytosing polyurethane nanoparticles by a ratiometric pH nanosensor. Adv. Biol. 5, 2000200. doi: 10.1002/ADBI.202000200 33724730

[B82] Wulff H. (2008). New light on the “Old”. Chloride Channel Blocker DIDS. J. Chem. Biol. 3, 399–401. doi: 10.1021/cb800140m 18642798

[B83] Yambire K. F. Rostosky C. Watanabe T. Pacheu-Grau D. Torres-Odio S. Sanchez-Guerrero A.. (2019). Impaired lysosomal acidification triggers iron deficiency and inflammation *in vivo* . Elife 8, 1–36. doi: 10.7554/ELIFE.51031 PMC6917501 31793879

[B84] Zhao J. Beyrakhova K. Liu Y. Alvarez C. P. Bueler S. A. Xu L.. (2017). Molecular basis for the binding and modulation of V-ATPase by a bacterial effector protein. PloS Pathog. 13, 1–21. doi: 10.1371/JOURNAL.PPAT.1006394 PMC5469503 28570695

[B85] Zuhlke L. J. Chb M. B. Fcpaeds D. C. H. Card C. Fesc M. P. H. Beaton A.. (2017). Group a streptococcus, acute rheumatic fever and rheumatic heart disease: Epidemiology and clinical considerations. Curr. Treat Options Cardio Med. 19, 1–23. doi: 10.1007/s11936-017-0513-y PMC5346434 28285457

