# Supplementary material for: Streptolysin S targets the sodium-bicarbonate cotransporter NBCn1 to induce inflammation and cytotoxicity in human keratinocytes during Group A Streptococcal infection
Source: Front Cell Infect Microbiol. 2022 Nov 1;12:1002230. doi: 10.3389/fcimb.2022.1002230 (PMC9663810; doi:10.3389/fcimb.2022.1002230)
Supplement: Supplementary file 1 [file DataSheet_1.zip › Data Sheet 1/SLS_Supplemental_Hammers_Frontiers_Revised_2.docx]

Supplementary Material


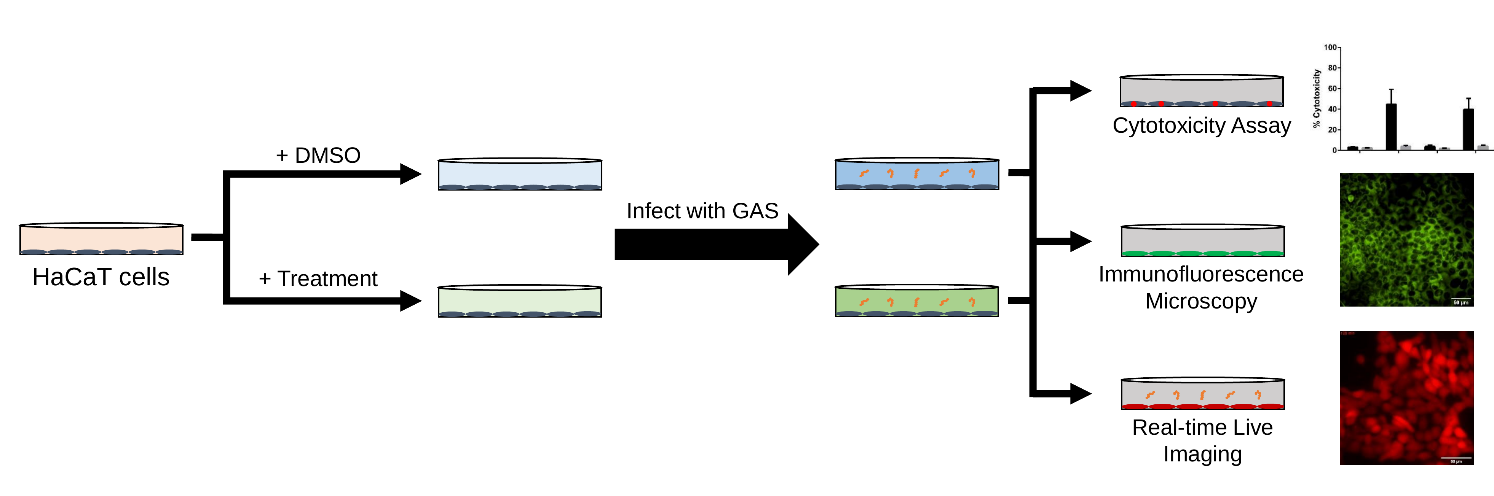


**Supplemental Figure 1: Summary of infection methods.** Immortalized human keratinocytes (HaCaT cells) were pre-treated with a chemical inhibitor or DMSO as a vehicle control. Keratinocytes were then infected with either wild-type, ΔsagA, or ΔsagA + sagA GAS at the specified MOI and time course, along with corresponding uninfected controls. Following the infections, a variety of measurements were performed: percent cytotoxicity was measured using an ethidium homodimer assay (top right), NF-κB activity was assessed using immunofluorescence microscopy (middle right), or pHrodo Red AM-loaded cells were infected and monitored in real time using live imaging (bottom right).


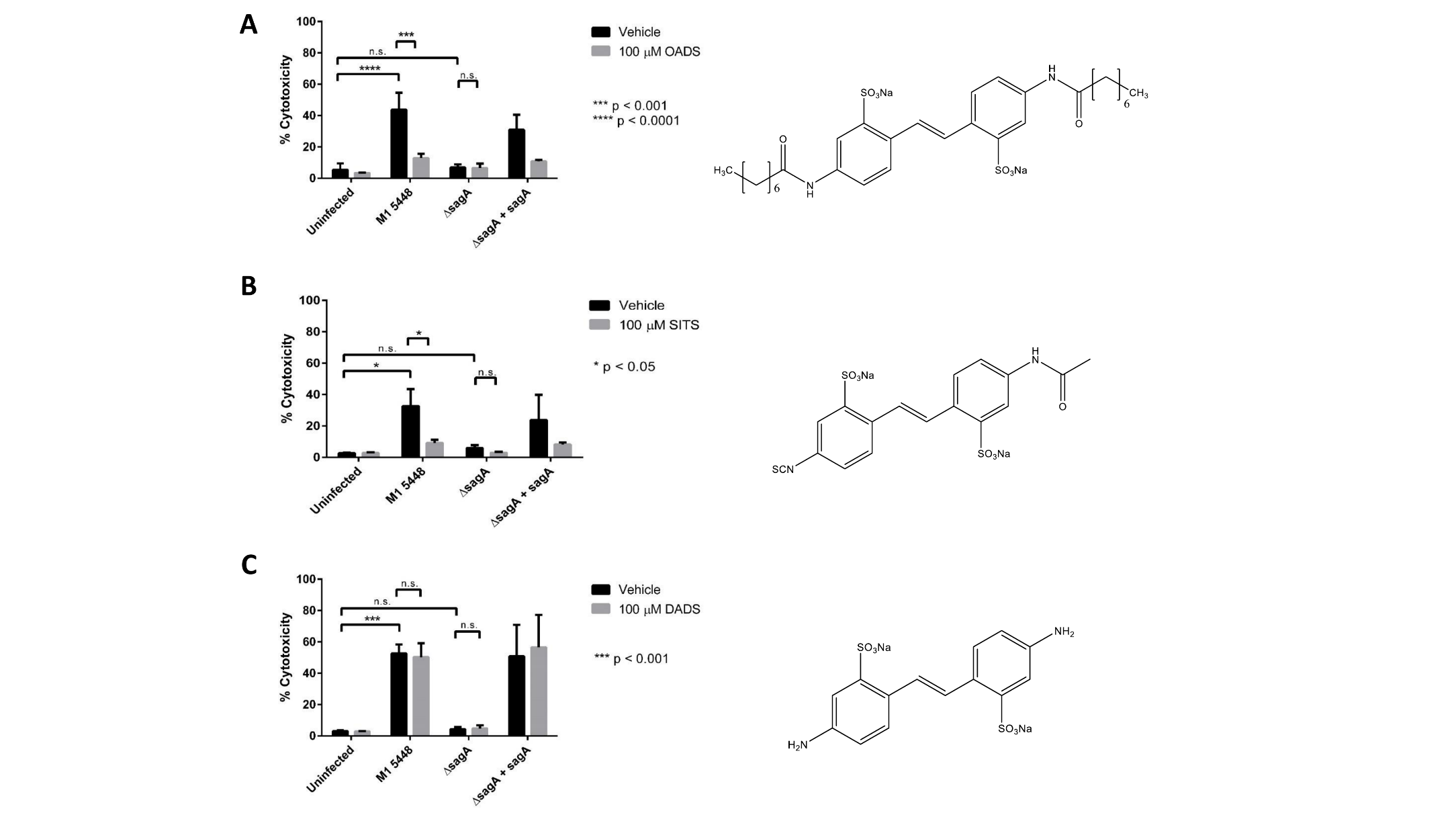


**Supplemental Figure 2: The isothiocyanate groups of DIDS impact its ability to inhibit SLS-mediated cytotoxicity.** Percent host cytotoxicity following 6 hour GAS infections of HaCaTs (MOI 10) pretreated for 1 hour with 100 µM each OADS (**A**), SITS (**B**), or DADS (**C**). Percent cytotoxicity was determined using an ethidium homodimer assay. DMSO was used as a vehicle control for all infections. Data are presented as mean ± sd, with n = 3 for each condition.


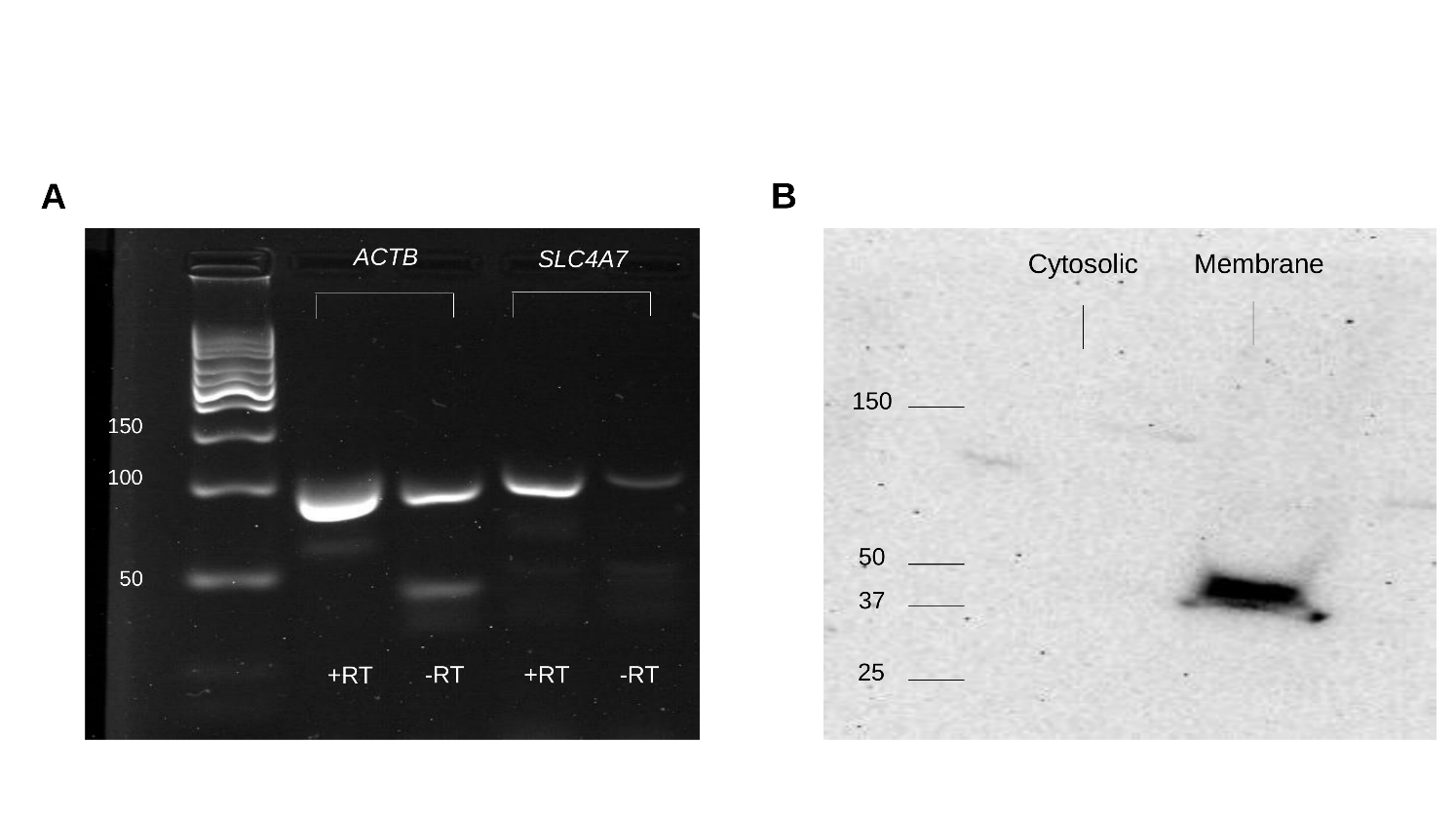


**Supplemental Figure 3: *SLC4A7* is expressed in HaCaT cells.** (**A**) RT-PCR data from cDNA generated from total HaCaT RNA. Beta actin (*ACTB*) was used as a housekeeping gene. Primer sequences were designed to span exon-exon junctions, and sequences were as follows: *ACTB*: Forward – 5’ GGATCAGCAAGCAGGAGTATG 3’, Reverse: 5’ AGAAAGGGTGTAACGCAACTAA 3’, annealing temperature 51° C, expected product size 96 bp. *SLC4A7*: Forward – 5’ GTGCCTGTATGTCTCCTGTAATC 3’, Reverse: 5’ TCCCAGTTAATGATGCTCCAAA 3’, annealing temperature 51° C, expected product size 105 bp. All PCR products were ran on a 3.5% agarose gel in TBE with a 50 bp ladder. 40 cycles of PCR were performed to sufficiently amplify the *SLC4A7* band when reverse transcriptase was present (+RT). Some nonspecific amplification was observed for both genes when reverse transcriptase was absent (-RT), but these bands were considerably weaker than the bands in the +RT conditions. (**B**) Western blot data from HaCaT lysates obtained using a hypoosmotic lysis buffer. The cytosolic fraction was subjected to SDS-PAGE and Western blotting alongside the membrane fraction that was isolated from the cell lysate using ultracentrifugation. The Rb α SLC4A7 antibody identified a single band in the membrane fraction of the cell lysate around 40 kDa. This represents a protein product corresponding to a portion of NBCn1 that is expressed in HaCaT cells under our cell culture conditions.


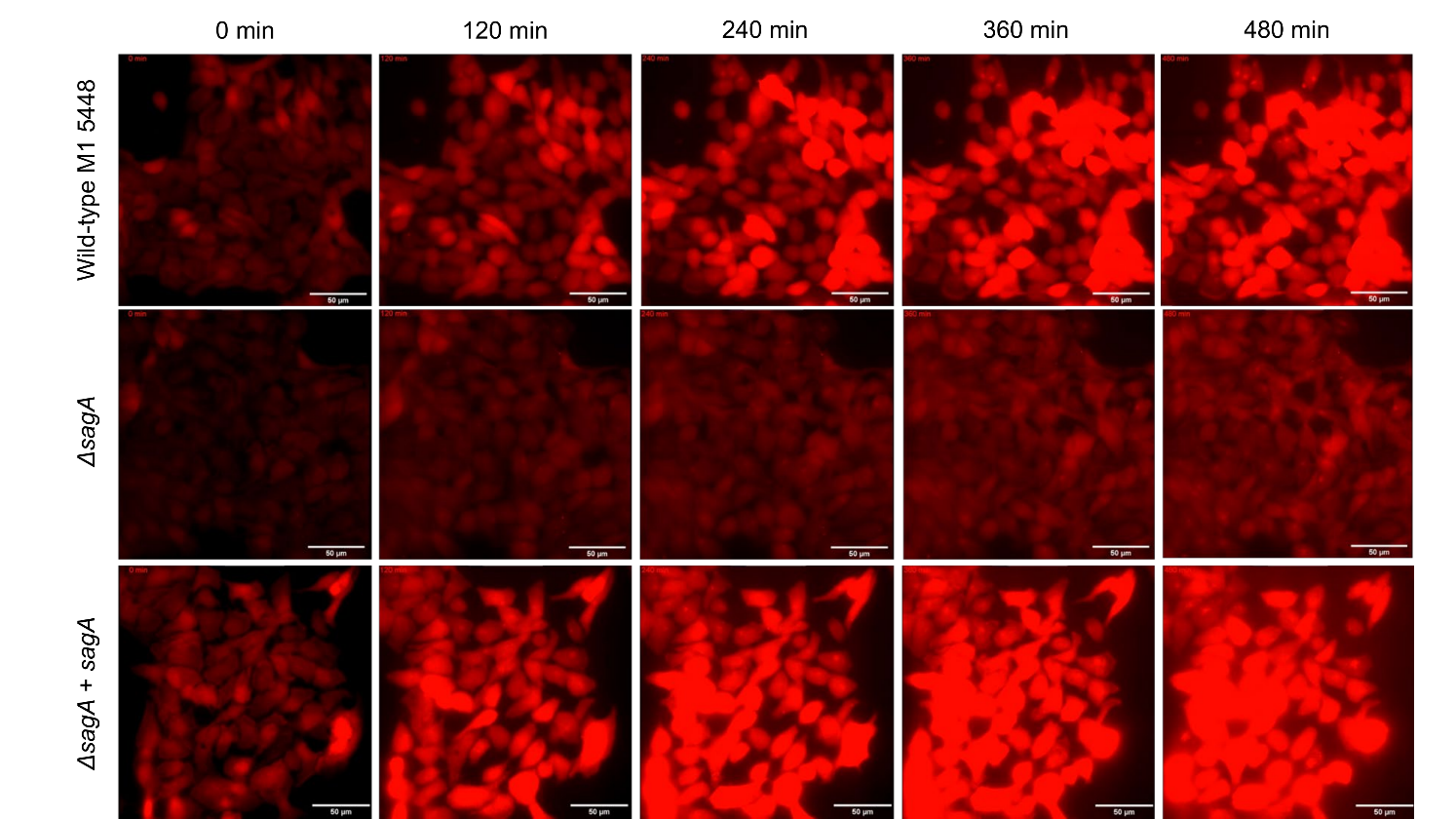


**Supplemental Figure 4: SLS treatment results in intracellular acidification of epithelial cells during GAS infection.**

HaCaT cells were loaded with pHrodo Red AM and were infected with either wild-type, *ΔsagA*, or *ΔsagA + sagA* GAS on a live imaging microscope at 37° C with 5% CO_2_. Fluorescence images were taken every 10 minutes for 8 hours. Frames for the specified time points are shown for each strain. Scale bar = 50 µm.


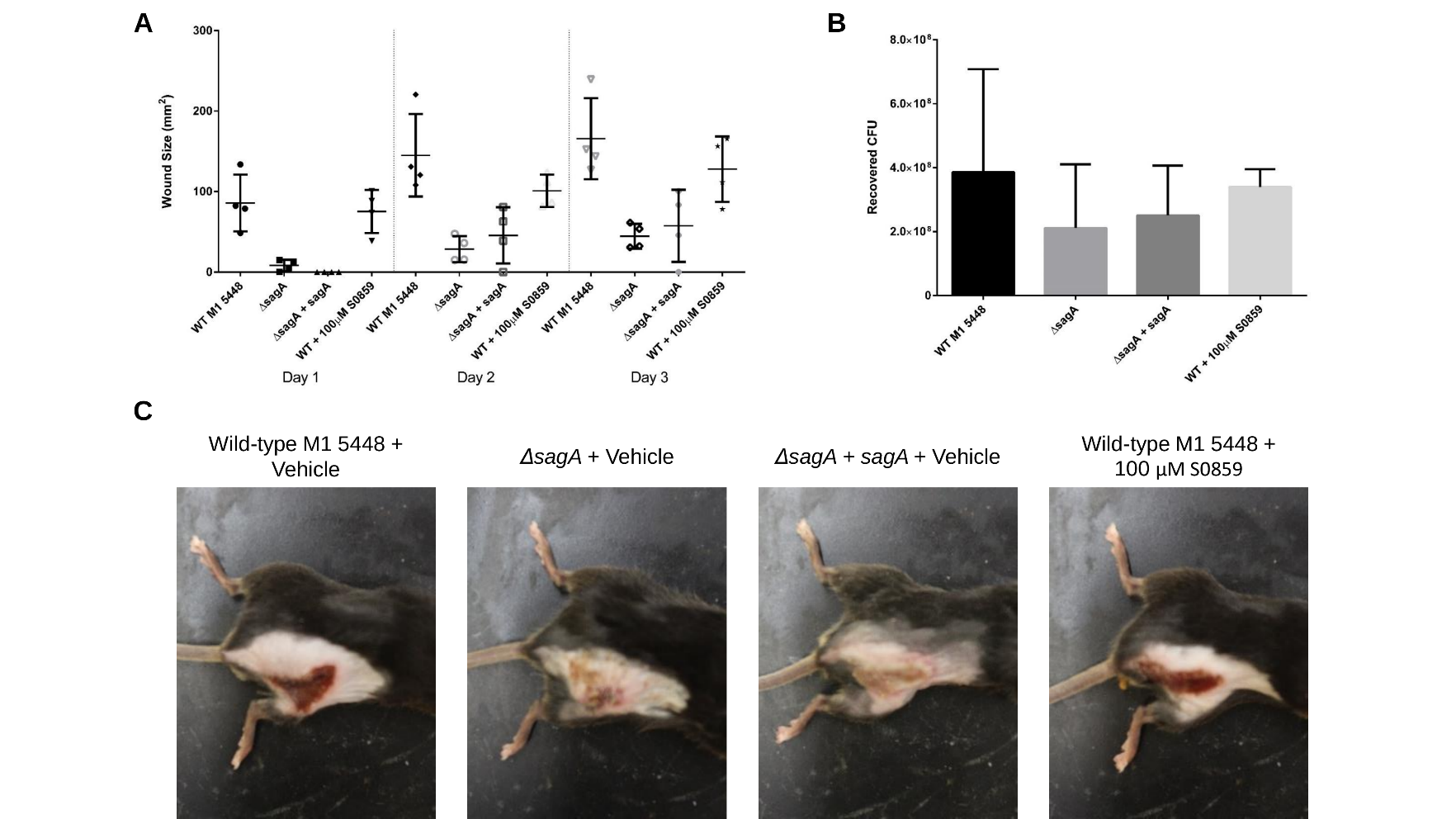


**Supplemental Figure 5: A single 100 µM S0859 treatment does not affect wound size in an *in vivo* model of GAS infection.**

C57BL/6 mice expressing the human plasminogen transgene were injected subcutaneously with wild-type M1 5448 GAS in the presence of 0.7% saline (vehicle) or 100 µM S0859, or the *ΔsagA* or *ΔsagA + sagA* strains in the presence of vehicle. Lesions were monitored for 72 hours, with measurements taken every 24 hours. After the endpoint of the infection, mice were sacrificed, and lesions were harvested for CFU recovery. (**A**) Wound size in mm^2^ measured every 24 hours over the course of the infection. (**B**) Recovered CFU from skin lesions taken after the infection. (**C**) Representative images of wounds from each group taken at 72 hours post-infection.

**Supplemental Video 1: Infection of pHrodo-loaded keratinocytes with wild-type GAS results in an increase in fluorescence intensity over time.** HaCaT cells were loaded with pHrodo Red AM and infected with wild-type M1 GAS at MOI 0.01, 37° C, 5% CO_2_. Images were taken in the pHrodo channel (**A**) and DIC channel (**B**) every 10 minutes for 8 hours. The scale bar represents 50 µM. The increase in fluorescence intensity corresponds to a decrease in pH_i_.

**Supplemental Video 2: Infection of pHrodo-loaded keratinocytes with *ΔsagA* GAS results in a minimal increase in fluorescence intensity over time.** HaCaT cells were loaded with pHrodo Red AM and infected with *ΔsagA* M1 GAS at MOI 0.01, 37° C, 5% CO_2_. Images were taken in the pHrodo channel (**A**) and DIC channel (**B**) every 10 minutes for 8 hours. The scale bar represents 50 µM. The minimal increase in fluorescence intensity suggests a minimal change in pH_i_.

**Supplemental Video 3: Infection of pHrodo-loaded keratinocytes with *ΔsagA* *+ sagA* GAS results in an increase in fluorescence intensity over time.** HaCaT cells were loaded with pHrodo Red AM and infected with *ΔsagA + sagA* M1 GAS at MOI 0.01, 37° C, 5% CO_2_. Images were taken in the pHrodo channel (**A**) and DIC channel (**B**) every 10 minutes for 8 hours. The scale bar represents 50 µM. The increase in fluorescence intensity corresponds to a decrease in pH_i_.

**Supplemental Video 4: S0859 treatment of keratinocytes induces rapid intracellular acidification in human keratinocytes during GAS infection.** HaCaT cells were loaded with pHrodo Red AM and treated with 100 µM S0859, and were then infected with wild-type M1 GAS at MOI 0.01, 37° C, 5% CO_2_. Images were taken in the pHrodo channel (**A**) and DIC channel (**B**) every 10 minutes for 8 hours. The scale bar represents 50 µM. The increase in fluorescence intensity corresponds to a decrease in pH_i_.

**
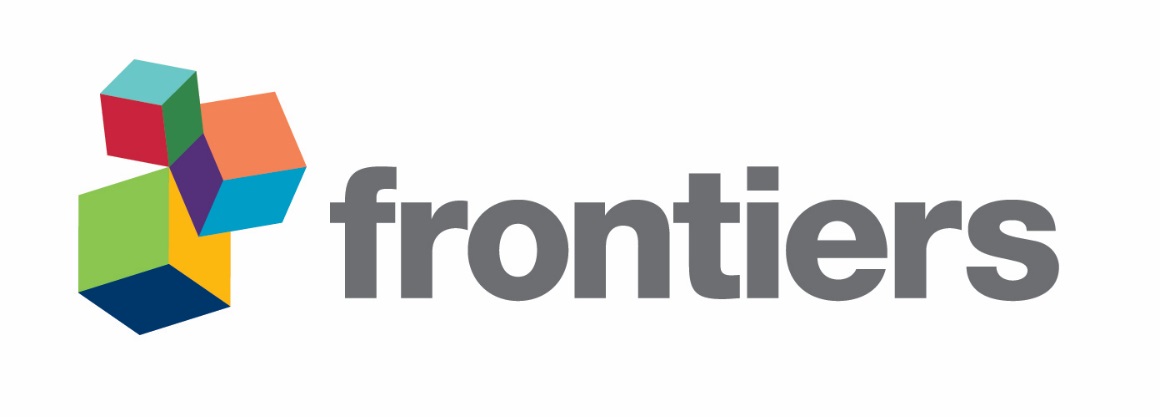
**
